# Supplementary material for: Tipping points in epithelial-mesenchymal lineages from single-cell transcriptomics data
Source: Biophys J. 2024 Mar 19;123(17):2849–59. doi: 10.1016/j.bpj.2024.03.021 (PMC11393678; doi:10.1016/j.bpj.2024.03.021)
Supplement: Document S1. Supporting methods, Tables S1 and S2, and Figures S1–S10 [file mmc1.pdf]

Biophysical Journal, Volume *123*

Supplemental information

Tipping points in epithelial-mesenchymal lineages from single-cell transcriptomics data

Manuel Barcenas, Federico Bocci, and Qing Nie

Supplementary Material

Tipping points in epithelial-mesenchymal lineages from single cell transcriptomics data

Running title: detecting bifurcations in scRNA-seq data

Manuel Barcenas[1,*], Federico Bocci[1,2,*,#], Qing Nie[1,2,#]

[1]Department of Mathematics and [2]NSF-Simons Center for Multiscale Cell Fate Research, University of California Irvine, 92697 Irvine, CA, USA
*These authors equally contributed to the research

[#]Authors for correspondence:
Qing Nie: qnie@uci.edu
Federico Bocci: fbocci@uci.edu

Supplementary Methods

1. Jacobian of the interacting RNA splicing model

Starting from eqs. (1), we rewrite the interacting RNA splicing model in terms of the functions f_i, g_i defined as:

$$\frac{dU_i}{dt} = f_i(\boldsymbol{U}, \boldsymbol{S}) = \sum_{j=1}^{N} a_{ij} S_j - U_i \quad (1a)$$

$$\frac{dS_i}{dt} = g_i(\boldsymbol{U}, \boldsymbol{S}) = U_i - \gamma_i S_i \quad (1b)$$

Where we have assumed $\beta_i = \beta = 1$ as explained in the main text, N is number of interacting RNA species, and the coefficients a_{ij} quantify the regulation of species j in species i. The Jacobian matrix is defined as:

$$J = \begin{pmatrix} \frac{\partial f_1}{\partial U_1} & \cdots & \frac{\partial f_1}{\partial U_N} & \frac{\partial f_1}{\partial S_1} & \cdots & \frac{\partial f_1}{\partial S_N} \\ \vdots & & \vdots & \vdots & & \vdots \\ \frac{\partial f_N}{\partial U_1} & \cdots & \frac{\partial f_N}{\partial U_N} & \frac{\partial f_N}{\partial S_1} & \cdots & \frac{\partial f_N}{\partial S_N} \\ \frac{\partial g_1}{\partial U_1} & \cdots & \frac{\partial g_1}{\partial U_N} & \frac{\partial g_1}{\partial S_1} & \cdots & \frac{\partial g_1}{\partial S_N} \\ \vdots & & \vdots & \vdots & & \vdots \\ \frac{\partial g_N}{\partial U_1} & \cdots & \frac{\partial g_N}{\partial U_N} & \frac{\partial g_N}{\partial S_1} & \cdots & \frac{\partial g_N}{\partial S_N} \end{pmatrix} = \begin{pmatrix} -1 & \cdots & 0 & a_{11} & \cdots & a_{1N} \\ \vdots & \ddots & \vdots & \vdots & & \vdots \\ 0 & \cdots & -1 & a_{N1} & \cdots & a_{NN} \\ 1 & \cdots & 0 & -\gamma_1 & \cdots & 0 \\ \vdots & \ddots & \vdots & \vdots & \ddots & \vdots \\ 0 & \cdots & 1 & 0 & \cdots & -\gamma_N \end{pmatrix}$$

Therefore, for a system of N interacting species, the Jacobian is a $2N \times 2N$ matrix whereby the top right quadrant, which corresponds to the derivative of the unspliced force field with respect to spliced species (highlighted in red box), assumes the interpretation of a gene regulatory network.

2. Generation of in silico data

2.1 Toggle switch

We generalize a minimal toggle switch design with two species (x, y) that mutually inhibit each other to include splicing dynamics while conserving the model's bistability. The temporal dynamics of unspliced and spliced versions of x and y (U_x, U_y, S_x, S_y) is modeled via stochastic ordinary differential equations:

$$\frac{dU_x}{dt} = \frac{\alpha}{1 + {S_y}^{\beta_1}} - \gamma U_x + \sigma\, dW(t) \quad (1a)$$

$$\frac{dU_y}{dt} = \frac{\alpha}{1 + {S_x}^{\beta_2}} - \gamma U_y \; + \sigma\, dW(t) \quad (1b)$$

$$\frac{dS_x}{dt} = \gamma U_x - S_x + \sigma\, dW(t) \quad (1c)$$

$$\frac{dS_y}{dt} = \gamma U_y - S_y + \sigma\, dW(t) \quad (1d)$$

Where α is a basal production rate, γ is a splicing rate coefficient, and β_1 , β_2 are Hill coefficients that determine the strength of feedback regulation. To simplify the model, α and γ are assumed to be equal for the two species. Furthermore, the degradation/dilution coefficient for spliced species S_x, S_y is assumed to be 1, which can be achieved by rescaling time. Finally, the noise $\sigma\, dW(t)$ is the product of an amplitude term σ and a random variable sampled from a normal distribution with standard deviation $\sqrt{\Delta t}$, where Δt is the timestep used for numerical solution. To induce a saddle node bifurcation, the parameter β_1 is gradually increased with time starting from $\beta_1 = 2$ to $\beta_1 = 15$. To generate the *in silico* dataset, we simulated $n = 1000$ trajectories of eqs. (1) using Euler-Maruyama starting from $t = 0$ (i.e., $\beta_1 = 2$) until $t = 200$ (i.e., $\beta_1 = 15$). From each independent simulation, a "cell" is sampled by taking the values of (U_x, U_y, S_x, S_y) at a randomly selected point along the trajectory. All other parameter values are fixed to $\alpha = 1$, $\beta_2 = 6$, $\gamma = 1$, $\sigma = 1$. All code was generated by the authors.

2.2 EMT circuit

We simulate epithelial-mesenchymal transition (EMT) with the EMT tristable circuit proposed by Tian and collaborators, which we previously generalized to include RNA splicing dynamics. This model uses ODEs to describe the dynamics of unspliced and spliced levels for: miR-34, miR-200, ZEB, SNAIL, external TGF-beta signal, cellular TGF-beta, E-cadherin, and N-cadherin. For each species, the unspliced equation includes basal transcription rate, feedback regulation from other species, and loss due to splicing; the spliced equation includes production due to splicing and degradation/dilution:

Intracellular TGF-beta (U_T, S_T):

$$\frac{dU_T}{dt} = k0_T + \frac{k_T}{1 + \left(\frac{S_{R_2}}{J_T}\right)^{n_{r2}}} - \beta U_T \quad (4a)$$

$$\frac{dS_T}{dt} = \beta U_T - kd_T S_T \quad (4b)$$

Snail mRNA (U_s, S_s) and protein (U_S, S_S):

$$\frac{dU_s}{dt} = k0_s + k_s \frac{\left(\frac{S_T + TGF0}{J_T}\right)^{n_t}}{1 + \left(\frac{S_T + TGF0}{J_T}\right)^{n_t}} - \beta U_s \quad (5a)$$

$$\frac{dS_s}{dt} = \beta U_s - kd_s S_s \quad (5b)$$

$$\frac{dU_S}{dt} = k0_s + k_S S_s \frac{1}{1 + \left(\frac{S_{R_3}}{J_S}\right)^{n_{r3}}} - \beta U_S \quad (6a)$$

$$\frac{dS_S}{dt} = \beta U_S - kd_S S_S \quad (6b)$$

miR-34 (U_{R3}, S_{R3}):

$$\frac{dU_{R3}}{dt} = k0_3 + \frac{k_3}{1 + \left(\frac{S_S}{J1_3}\right)^{n_S} + \left(\frac{S_Z}{J2_3}\right)^{n_Z}} - \beta U_{R3} \quad (7a)$$

$$\frac{dS_{R3}}{dt} = \beta U_{R3} - kd_3 S_{R3} \quad (7b)$$

Zeb mRNA (U_z, S_z) and protein (U_Z, S_Z):

$$\frac{dU_z}{dt} = k0_z + k_z \frac{\left(\frac{S_S}{J_Z}\right)^{n_s}}{1 + \left(\frac{S_S}{J_Z}\right)^{n_s}} - \beta U_z \quad (8a)$$

$$\frac{dS_z}{dt} = \beta U_z - kd_z S_z \quad (8b)$$

$$\frac{dU_Z}{dt} = k0_Z + k_Z S_z \frac{1}{1 + \left(\frac{S_{R_2}}{J_Z}\right)^{n_{r2}}} - \beta U_Z \quad (9a)$$

$$\frac{dS_Z}{dt} = \beta U_Z - kd_Z S_Z \quad (9b)$$

miR-200 (U_{R2}, S_{R2}):

$$\frac{dU_{R2}}{dt} = k0_2 + \frac{k_2}{1 + \left(\frac{S_S}{J1_2}\right)^{n_S} + \left(\frac{S_Z}{J2_2}\right)^{n_Z}} - \beta U_{R2} \quad (10a)$$

$$\frac{dS_{R2}}{dt} = \beta U_{R2} - kd_2 S_{R2} \quad (10b)$$

E-cadherin (U_E, S_E):

$$\frac{dU_E}{dt} = k0_E + \frac{k_{E1}}{1 + \left(\frac{S_S}{J1_E}\right)^{n_S}} + \frac{k_{E2}}{1 + \left(\frac{S_Z}{J2_E}\right)^{n_Z}} - \beta U_E \quad (11a)$$

$$\frac{dS_E}{dt} = \beta U_E - kd_E S_E \quad (11b)$$

N-cadherin (U_N, S_N):

$$\frac{dU_N}{dt} = k0_N + k_{N1} \frac{\left(\frac{S_S}{J1_N}\right)^{n_S}}{1 + \left(\frac{S_S}{J1_N}\right)^{n_S}} + k_{N2} \frac{\left(\frac{S_Z}{J2_N}\right)^{n_Z}}{1 + \left(\frac{S_Z}{J2_N}\right)^{n_Z}} - \beta U_N \quad (12a)$$

$$\frac{dS_N}{dt} = \beta U_N - kd_N S_N \quad (12b)$$

Parameter values were taken from the original model. The splicing rate coefficient (β) is assumed to be rescaled to unit value. The system of ODEs was simulated numerically using Euler-Maruyama algorithm. All code was generated by the authors.

2.3 Trifurcating circuit

We simulate $n = 2000$ cells in the trifurcating circuit consisting of 8 mutually regulating genes using the BoolODE package. All circuit and simulation parameter were fixed to their original value. The output of BoolODE consists of two gene expression matrices of size 8x2000 corresponding to two layers of expression, interpreted here as the unspliced and spliced RNA counts, and the simulation time associated to each cell.

3. Calculation and visualization of instability score

The instability score of a single cell along a lineage is defined as the average number of positive eigenvalues associated with the cell. Since a cell can appear in multiple bins along pseudotime axis (see previous section), the score averages the number of positive eigenvalues in each pseudotime bin where the cell is found. When multiple lineages are present in the dataset (see Fig. 3), the score is first computed within each individual lineage and then averaged if the cell belongs to multiple lineages. Finally, the instability score is plotted and overlayed on RNA velocity using scVelo's velocity_embedding_stream() function.

4. Data preparation and clustering

The unspliced and spliced scRNA-seq RNA counts for both OVCA420 and A549 datasets were taken from Cook and collaborators, and later filtered and normalized following standard procedures using scanpy. The n=50 top genes were used for Jacobian inference and downstream GRN analysis. Diffusion pseudotime for the OVCA420 dataset was computed using scanpy's built-in diffusion pseudotime function with default parameters, whereby the root cell for pseudotime calculation (i.e., the cell at pseudotime t=0) was set as a randomly chosen cell from the cell group sampled at t=0 days. Velocity pseudotime for the A549 dataset was computed using scVelo's built in function with default parameters.

5. Gene selection criteria

Scanpy's highly variable feature function was used to select the top 50 differentially expressed genes for both the OVCA420 and A549 cell line datasets. The set of top 50 genes used in Figure 4 are presented in supplementary table 2. Furthermore, the number of differentially expressed genes selected via scanpy's highly variable feature function was varied (N=25, 50, 75, 100) to test the method robustness (supplementary figure S5). Finally, we test the method using a user-curated list of epithelial and mesenchymal genes recovered from literature. First, we used scanpy's highly variable feature function to select the top 2000 genes in the dataset to ensure that low-count genes were filtered out. Then, we subset the dataset and kept only the epithelial and mesenchymal genes, resulting in 24 epithelial genes and 60 mesenchymal genes (supplementary table 3).

6. Inference of Jacobian matrix and gene regulatory network

The input for Jacobian inference includes the unspliced and spliced RNA counts as well as the pseudotime value for each cell defined in the previous section. First, cells were binned within intervals along the pseudotime coordinate. A width parameter (w) sets how wide the pseudotime

bins are, whereas an increment parameter (inc) sets the forward step taken along the pseudotime axis after each inference iteration. Considering that diffusion pseudotime is typically bound in [0,1], we selected w=0.1, inc=0.05, leading to 20 equally spaced points along the lineage. We stress that a "correct" value for these parameters cannot be established a priori, and it is the result of trial and error. Second, all cells within each bin are used to infer the Jacobian matrix and gene regulatory network following the strategy highlighted in the main method section "*Identification of tipping points along lineage*".

7. Comparison with Dynamo

While our method computes an average Jacobian matrix within a specified reaction coordinate window (diffusion pseudotime in the case of Fig. 4), Dynamo reconstructs a Jacobian matrix associated to individual cells. To set up a fair comparison, we average the Jacobian matrix calculated by Dynamo over the same pseudotime inference window. Initially, we run Dynamo's Vectorfield function to compute a cell-specific Jacobian matrix. Then, we group cells based on their position along the diffusion pseudotime axis, and average Dynamo's cell-specific Jacobian matrices. We compare the methods based on different criteria for gene set selection. First, we let Dynamo's preprocessing pipeline select the genes for Jacobian inference; second, we filter the dataset object with the genes used in our calculation before running Dynamo's Vectorfield function. Third, we provide the gene set used in our calculation as input for Dynamo's Vectorfield function. In all three cases, the stability along the EMT lineage is evaluated based on both (1) The number of positive eigenvalues and (2) the value of the largest eigenvalue.

8. Community detection in gene regulatory network

GRN graph objects were generated using the NetworkX package. GRN communities were calculated using NetworkX's built-in functions girvan_newman() and greedy_modularity_communities() using default parameters.

9. Code availability

All scripts were generated in by the authors and are available at https://github.com/barcenasmanuel/Detecting_bifurcations_in_scRNA_seq_data.

OVCA420	TSPAN1, ATP1B1, LAMC2, CENPF, GATA3, PLAU, FGF19, LIMA1, KRT81, KRT6A, HSP90B1, NFKBIA, SNAPC1, C15orf48, NQO1, MMP2, CCL5, TOP2A, BCAS3, KPNA2, SIPA1L3, CCDC88A, SERPINE2, ID2, CENPA, ANXA4, DHRS9, TGM2, AURKA, MMP9, CBR1, CBR3, LGALS1, MYLK, SPTSSB, TPRG1, C4orf26, ANXA3, ANXA10, FGF1, CDH6, FST, TFPI2, PODXL, HSPB1, SLC7A2, LPAR1, AMBP, TNC, SAT1

Supplementary table 1. List of top 50 genes used for stability inference in in OVCA420 cell line (Figure 4).

Epithelial geneset	ALDH1A3, AREG, DST, CD9, CDH1, EMP1, EPAS1, EREG, KRT19, ABLIM1, MITF, ATP8B1, PKP2, PPL, NR2F2, TPD52L1, MAP7, ARHGAP29, TJP2, HS3ST1, TSPAN1, SYNE2, EHF, GULP1
Mesenchymal geneset	ACTN1, BMPR2, BPGM, CALD1, CD59, CDH2, COL4A1, COL4A2, ELK3, EPHB2, ETS2, FN1, HMOX1, JARID2, LAMC2, MMP2, MMP9, MYO10, NEDD9, NT5E, SERPINE1, SERPINE2, PODXL, PTHLH, PTPRK, RALA, SKIL, TGFB1, TGFBI, TGM2, THBS1, TPM1, TPM4, TUFT1, TFPI2, HMGA2, INPP4B, PDLIM7, TP53I3, MICAL2, DLC1, SEMA3C, DAAM1, PALLD, TMCC1, PLEK2, DSE, LMCD1, CHST11, ANGPTL4, GALNT10, PDGFC, PMEPA1, SMURF2, FHOD3, LBH, MAP1LC3B, MBOAT2, AMIGO2, VGLL3

Supplementary Table 2. List of epithelial and mesenchymal genes used to compute mesenchymal scores (Fig. 4B-C and 5B-C) and the Jacobian inference based on the EMT gene signature.

Supplementary Figures

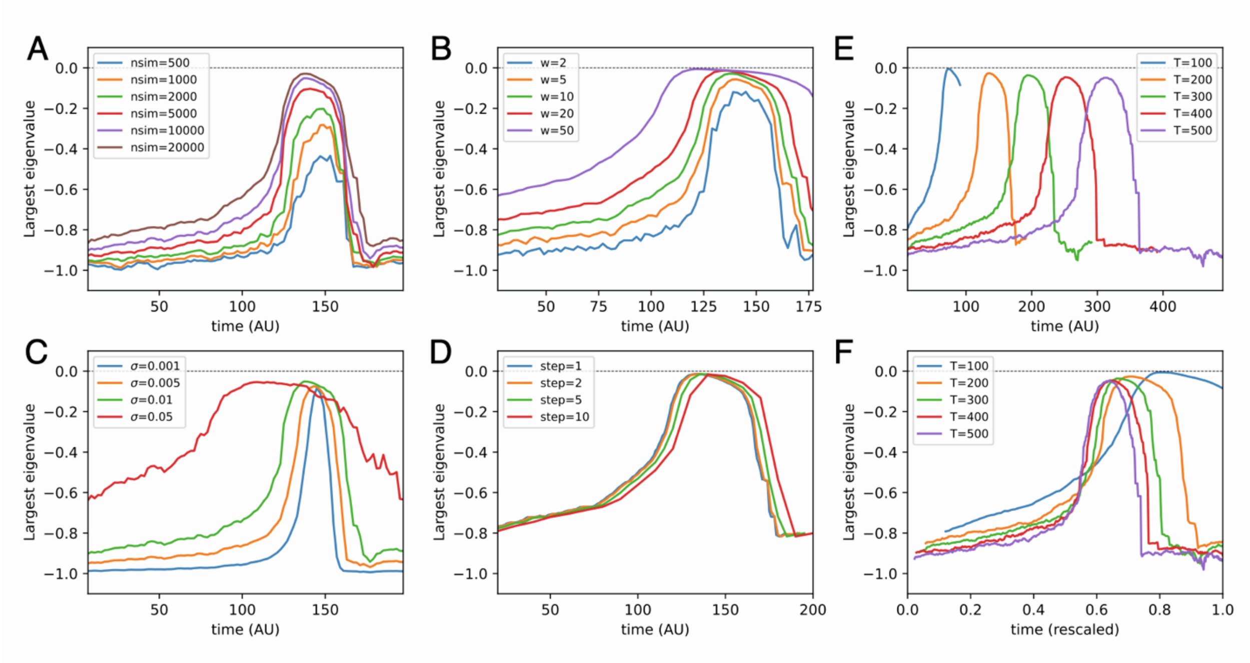

Supplementary figure S1. Supplementary analysis of the toggle switch circuit. (A) *Largest eigenvalue inferred in the toggle switch Jacobian matrix as a function of simulation time for increasing number of sampled cells.* ***(B)*** *Same as (A) for varying width of the inference window parameter.* ***(C)*** *Same as (A) for varying levels of noise amplitude.* ***(D)*** *Same as (A) for varying increment step of the inference window parameter.* ***(E)*** *Largest eigenvalue of the toggle switch Jacobian matrix as a function of time for increasing simulation length. The shortest simulation length (T=100 AU, blue curve) is not sufficient for cells to complete the saddle-node transition.* ***(F)*** *Same as (E) when renormalizing time (x-axis). The toggle switch undergoes the saddle-node transition at similar rescaled time for all simulation length except T=100 AU. Foe each panel, default parameters are same as in main Figure 2E-F (nsim=2000, σ=0.005, w=10, step=2, T=200 AU) except for the parameter that is varied in the specific panel.*

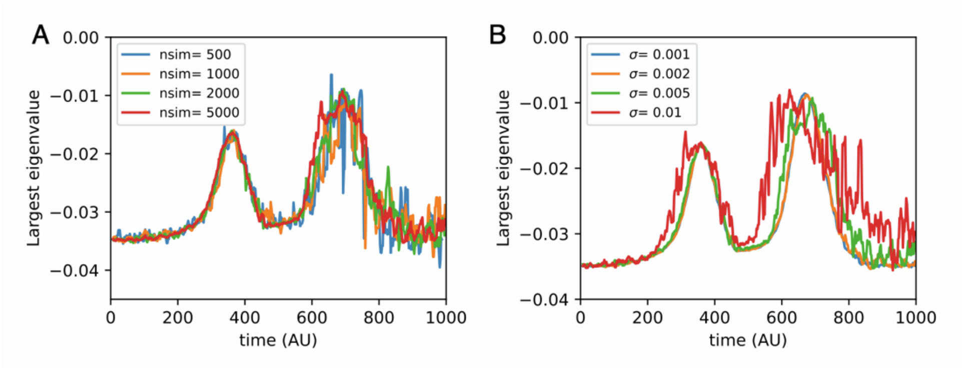

Supplementary figure S2. Supplementary analysis of the EMT circuit. (A) *Largest eigenvalue inferred in the EMT Jacobian matrix as a function of simulation time for increasing number of sampled cells.* ***(B)*** *Same as (A) for varying levels of noise amplitude. Foe each panel, default parameters are same as in main Figure 2G-H (nsim=2000, σ=0.005, w=10, step=2, T=1000 AU) except for the parameter that is varied in the specific panel.*

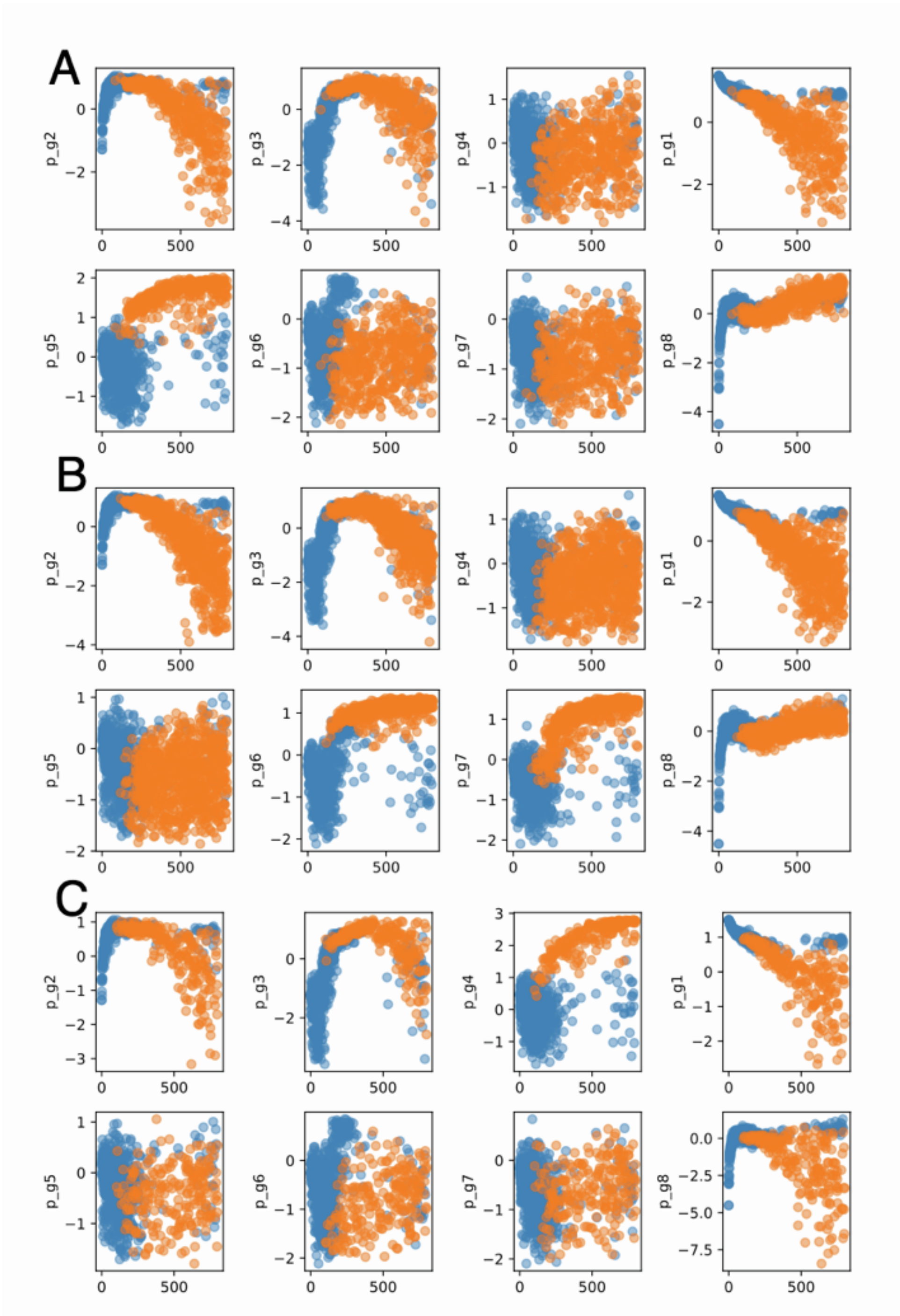

Supplementary figure S3. Supplementary analysis of the trifurcating circuit. (A) *Dynamics of the 8 spliced variable in the S-T1 transition in the trifurcating circuit. Each dot represents a simulated cell plotted against BoolODE simulation time (x-axis). Orange and blue coloring indicates cells belonging to the S and T1 clusters, respectively.* ***(B-C)*** *Same as (A) for the S-T2 and S-T3 transitions, respectively.*

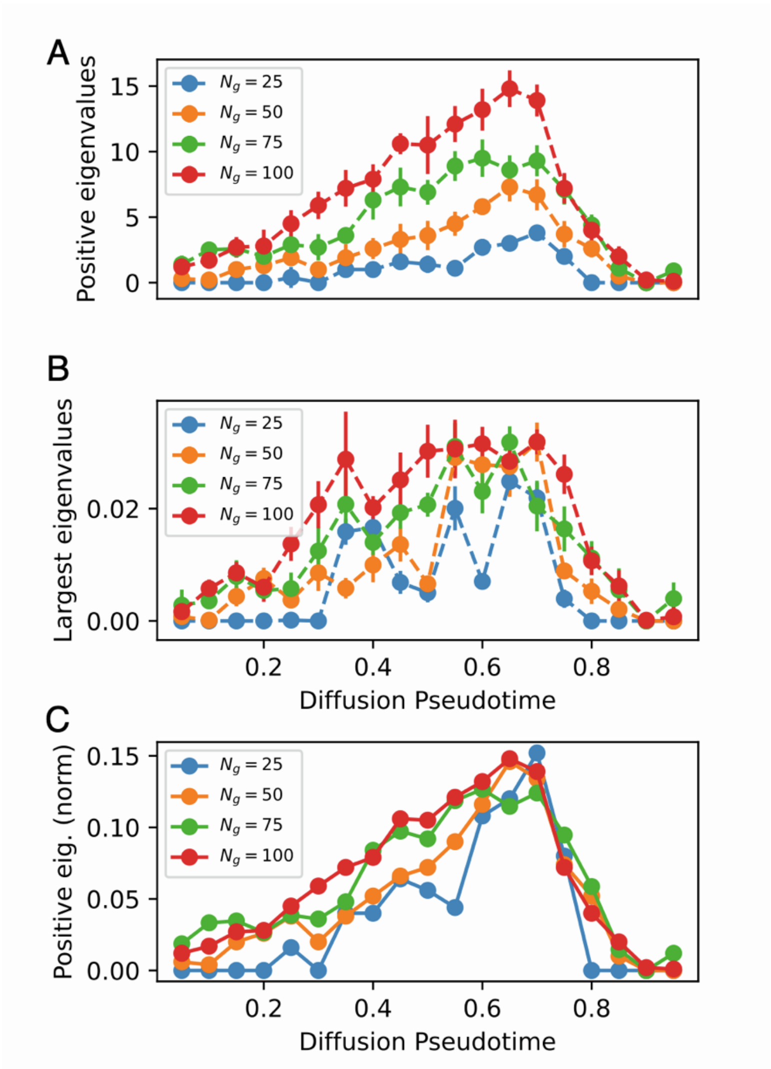

Supplementary figure S4. Method robustness to variation of the number of genes. (A) *Number of positive Jacobian eigenvalues as a function of diffusion pseudotime in the OVCA420 cell line dataset for increasing number of top genes selected for Jacobian inference.* ***(B)*** *Largest Jacobian eigenvalue as a function of pseudotime for increasing number of top genes.* ***(C)*** *Number of positive eigenvalues normalized by the total number of selected genes.*

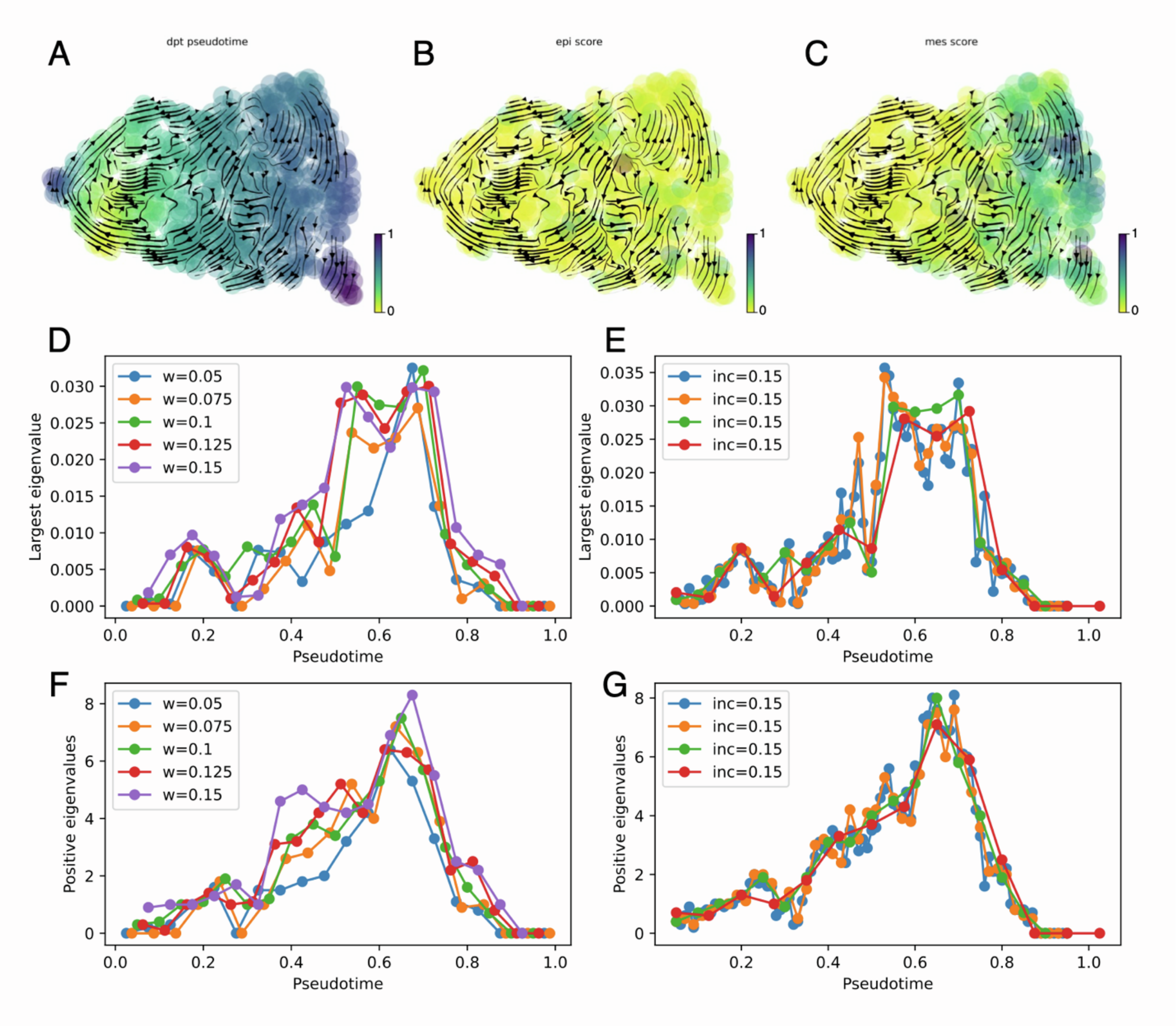

Supplementary figure S5. Supplementary analysis of the OVCA420 dataset. (A-B-C) *Diffusion pseudotime (A), epithelia score (B), and mesenchymal score (C) of OVCA420 cells overlayed on UMAP and RNA velocity.* ***(D)*** *Largest eigenvalue as a function of diffusion pseudotime. Different curves highlight different values of inference window width expressed in pseudotime units.* ***(E)*** *Largest eigenvalue as a function of diffusion pseudotime upon variation of inference window increment, expressed in pseudotime units.* ***(F-G)*** *Same as (D-E) for number of positive eigenvalues as a function of pseudotime.*

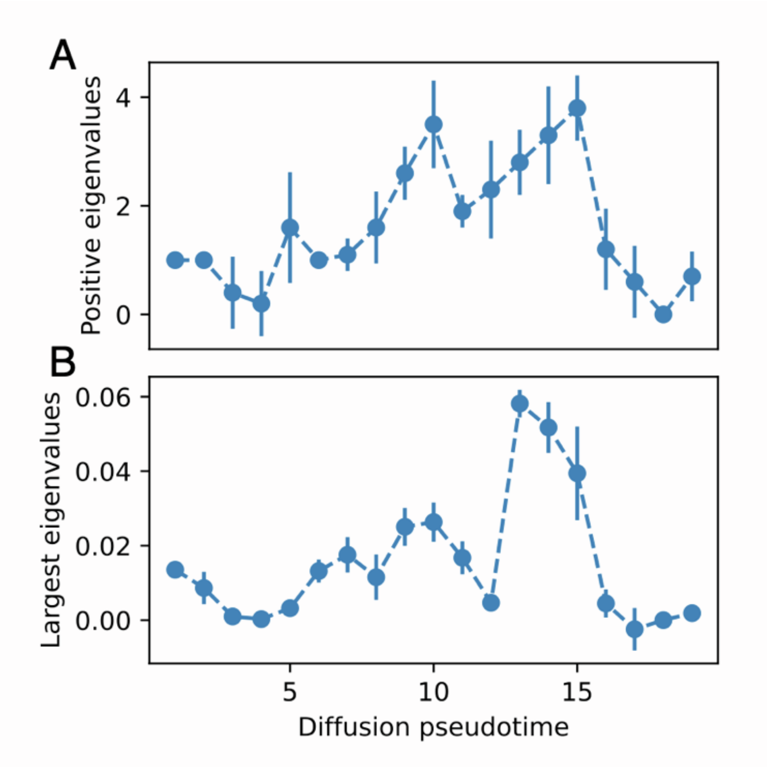

Supplementary figure S6. Jacobian inference based on EMT gene set. (A-B) *Number of positive eigenvalues (A) and largest Jacobian eigenvalue (B) in the OVCA420 cell line dataset as a function of diffusion pseudotime when a set of epithelial and mesenchymal genes is used for Jacobian inference.*

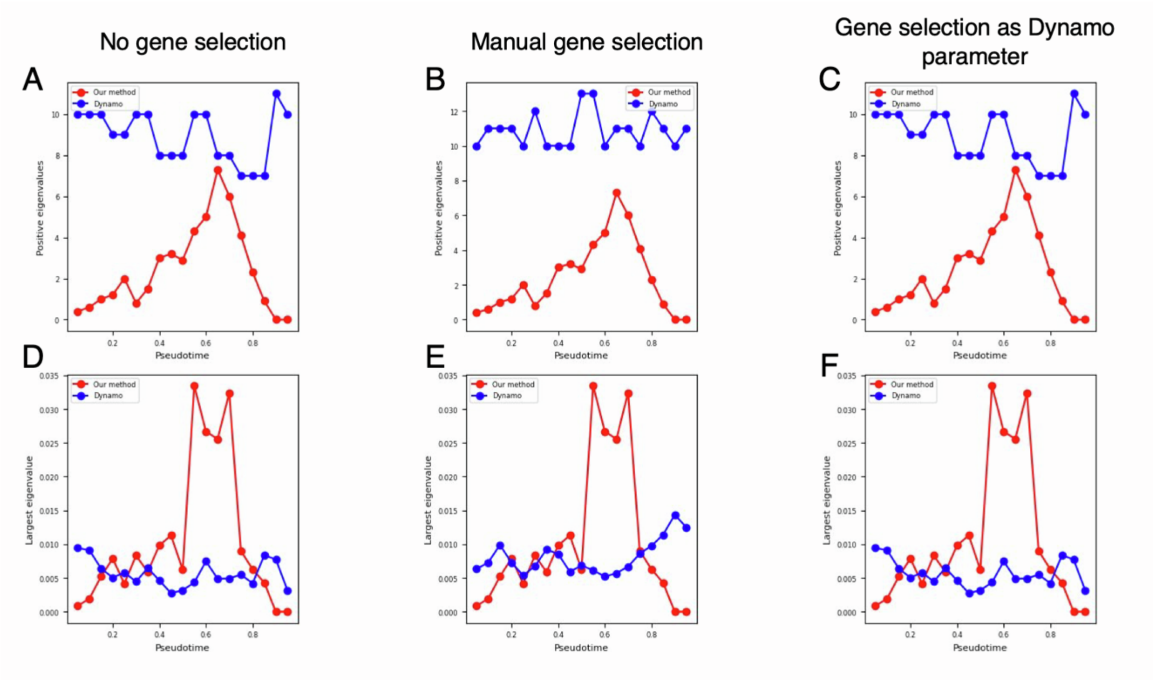

Supplementary figure S7. Comparison with Dynamo. (A-B) *Comparison for number of positive eigenvalues (A) and largest Jacobian eigenvalue (B) in the OVCA420 cell line dataset as a function of diffusion pseudotime between our method (red) and Dynamo (blue) when default criteria for Dynamo's gene selection. (C-D) Same as (A-B) when the dataset is manually filtered to include the top 50 genes used in our analysis before Dynamo is run. (E-F) Same as (A-B) when the list of top 50 genes used in our calculation is passed to Dynamo's Vectorfield function.*

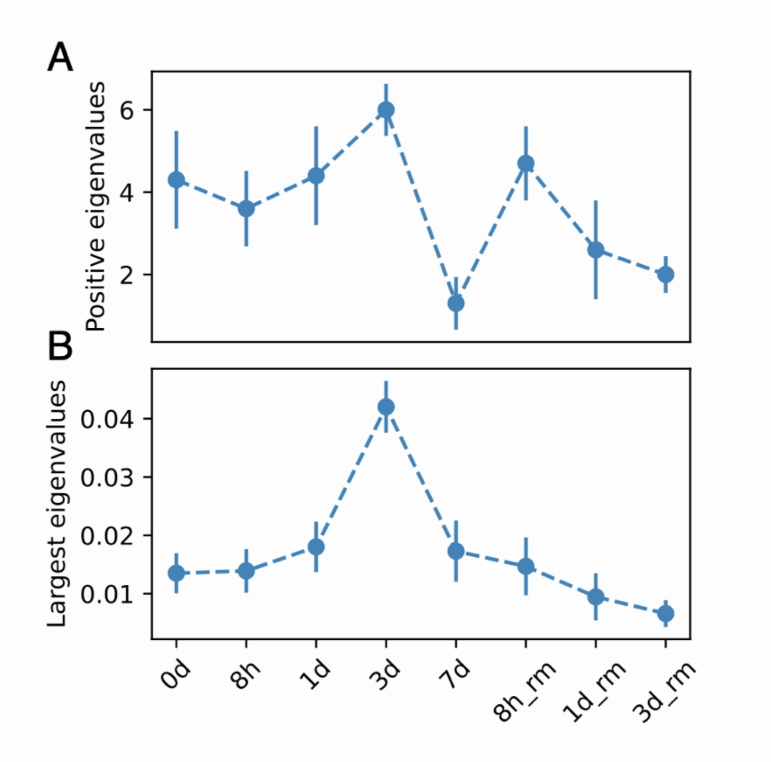

Supplementary figure S8. Jacobian inference based on time course points. (A-B) *Number of positive eigenvalues (A) and largest Jacobian eigenvalue (B) in the OVCA420 cell line dataset when using the time points if the time course for Jacobian inference.*

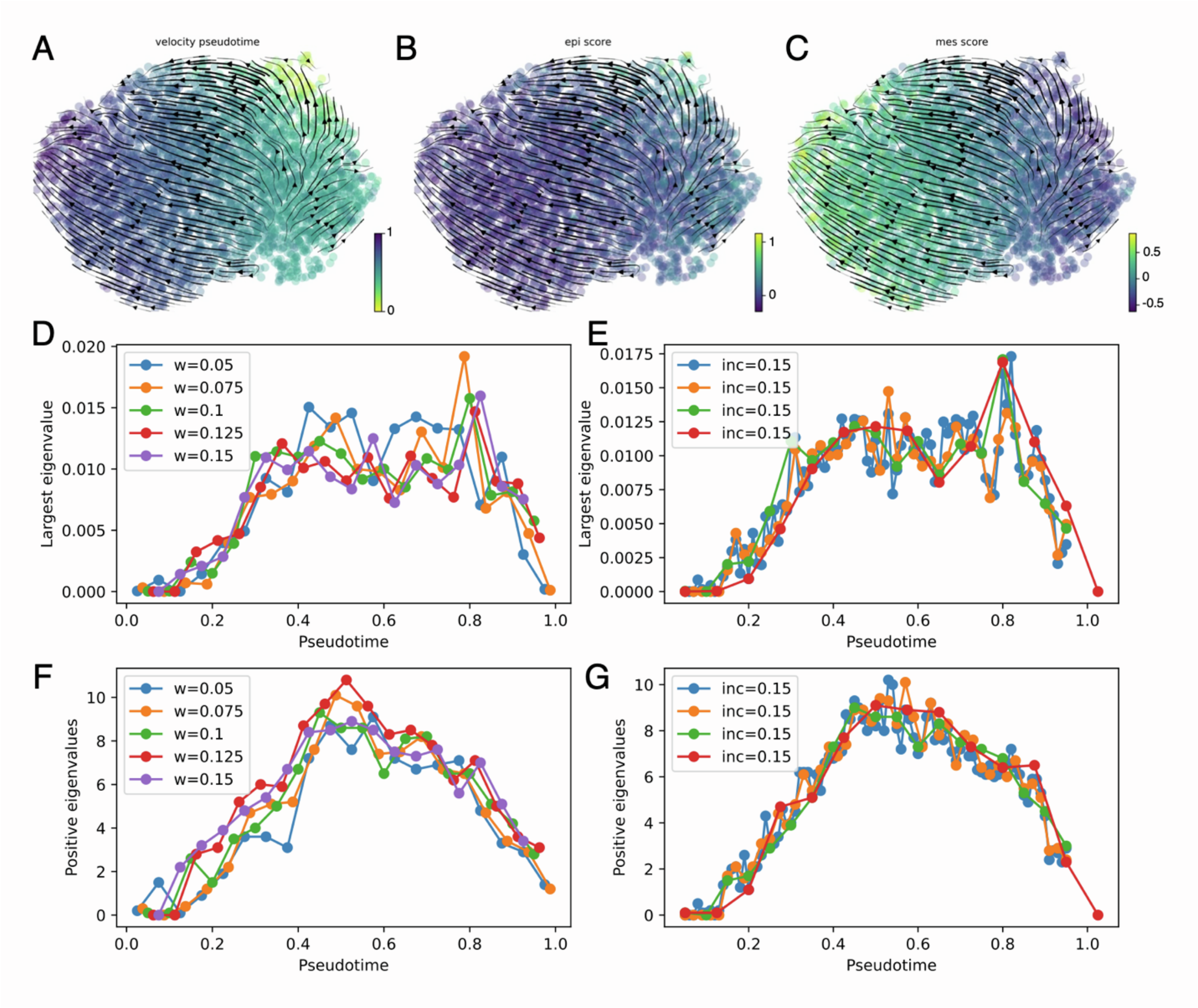

Supplementary figure S9. Supplementary analysis of the A549 dataset. (A-B-C) *Diffusion pseudotime (A), epithelia score (B), and mesenchymal score (C) of A549 cells overlayed on UMAP and RNA velocity.* ***(D)*** *Largest eigenvalue as a function of diffusion pseudotime. Different curves highlight different values of inference window width expressed in pseudotime units.* ***(E)*** *Largest eigenvalue as a function of diffusion pseudotime upon variation of inference window increment, expressed in pseudotime units.* ***(F-G)*** *Same as (D-E) for number of positive eigenvalues as a function of pseudotime.*

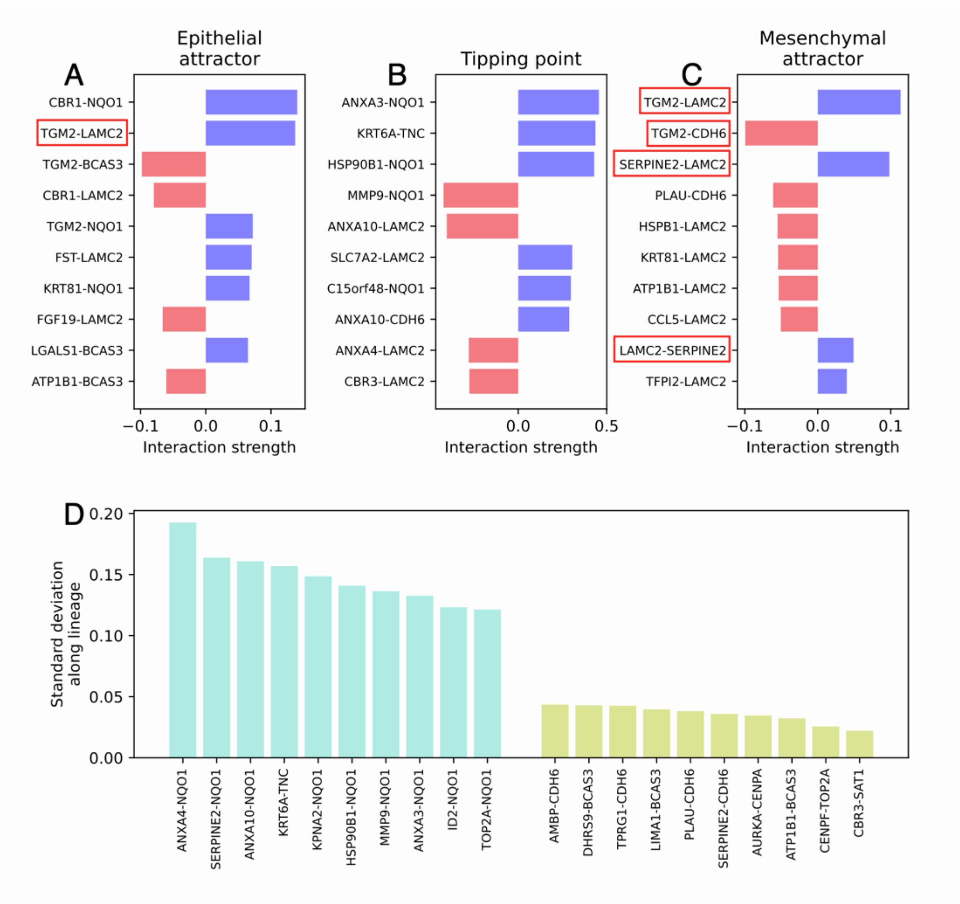

Supplementary figure S10. Statistics of gene-gene interactions in OVCA420 cell line. *(A-B-C) The top 10 strongest gene-gene interactions inferred in the pseudotime points corresponding to the epithelial attractor (as in Fig. 5B), tipping point (Fig. 5D), and mesenchymal attractor (Fig. 5F). Red boxes highlight connections between known epithelial (CHD6) and mesenchymal (TGM2, LAMC2, SERPINE2) genes showing that mesenchymal genes tend to positively regulate each other while inhibiting CDH6. (D) Statistics of the gene-gene interactions with larger (left, cyan) and lowest (right, yellow) standard deviation along the lineage.*
