# Supplementary material for: Tipping points in epithelial-mesenchymal lineages from single-cell transcriptomics data
Source: Biophys J. 2024 Mar 19;123(17):2849–59. doi: 10.1016/j.bpj.2024.03.021 (PMC11393678; doi:10.1016/j.bpj.2024.03.021)
Supplement: Document S2. Article plus supporting material [file mmc2.pdf]

Biophysical Journal
Article

Biophysical Society

Tipping points in epithelial-mesenchymal lineages from single-cell transcriptomics data

Manuel Barcenas,[1] Federico Bocci,[1,2,*] and Qing Nie[1,2,*]
[1]Department of Mathematics, University of California Irvine, Irvine, California and [2]NSF-Simons Center for Multiscale Cell Fate Research, University of California Irvine, Irvine, California

ABSTRACT Understanding cell fate decision-making during complex biological processes is an open challenge that is now aided by high-resolution single-cell sequencing technologies. Specifically, it remains challenging to identify and characterize transition states corresponding to "tipping points" whereby cells commit to new cell states. Here, we present a computational method that takes advantage of single-cell transcriptomics data to infer the stability and gene regulatory networks (GRNs) along cell lineages. Our method uses the unspliced and spliced counts from single-cell RNA sequencing data and cell ordering along lineage trajectories to train an RNA splicing multivariate model, from which cell-state stability along the lineage is inferred based on spectral analysis of the model's Jacobian matrix. Moreover, the model infers the RNA cross-species interactions resulting in GRNs and their variation along the cell lineage. When applied to epithelial-mesenchymal transition in ovarian and lung cancer-derived cell lines, our model predicts a saddle-node transition between the epithelial and mesenchymal states passing through an unstable, intermediate cell state. Furthermore, we show that the underlying GRN controlling epithelial-mesenchymal transition rearranges during the transition, resulting in denser and less modular networks in the intermediate state. Overall, our method represents a flexible tool to study cell lineages with a combination of theory-driven modeling and single-cell transcriptomics data.

SIGNIFICANCE Single-cell sequencing technologies offer unprecedented opportunities to inspect the mechanisms of cell fate commitment along cell lineages, i.e., transition processes whereby cells abandon an initial cell state and transition toward a new one. Here, we develop a computational model trained on single-cell RNA transcriptomics data to study cell stability during these transition processes and identify the tipping points whereby cells commit to new cellular states. Moreover, our model allows us to inspect intracellular gene regulation, thus offering a comprehensive picture of the change in the transcriptional dynamics during epithelial-mesenchymal transition through intermediate cell states.

INTRODUCTION

Single-cell sequencing technologies enable us now to closely monitor and dissect cell fate within individual cells, providing opportunities to study the cell's decision-making during cell fate commitment events. A key biological example of cell fate transition is the epithelial-mesenchymal transition (EMT), a *trans*-differentiation process whereby epithelial cells lose cell-cell adhesion while gaining motile traits (1). EMT is tightly controlled by gene regulatory networks (GRNs) including mesenchymal transcription factors and epithelial noncoding RNAs that have been previously studied using both mathematical modeling and data-driven inference methods (2,3). Specifically, mathematical modeling of the core EMT regulatory circuit suggests that EMT can be interpreted via one or multiple saddle-node transitions on an epithelial-mesenchymal landscape, whereby an initially stable cell state (e.g., epithelial) is destabilized, and cells travel through an instability before reaching the next stable state (e.g., mesenchymal) (4–6). The interpretation of cell fate transitions as pathways in an underlying complex landscape has been embraced in the biological community for several decades, first through the concept of the Waddington landscape whereby a cell navigates valleys (cell types) and ridges (transition areas) akin to a rolling marble (7,8). More recently, the Waddington landscape was quantified through mathematical modeling and stochastic simulations

Submitted December 11, 2023, and accepted for publication March 15, 2024.

*Correspondence: fbocci@uci.edu or qnie@uci.edu

Manuel Barcenas and Federico Bocci contributed equally to this work.

Editor: Jianhua Xing.
https://doi.org/10.1016/j.bpj.2024.03.021

© 2024 Biophysical Society.
This is an open access article under the CC BY license (http://creativecommons.org/licenses/by/4.0/).

in a variety of biological contexts, including EMT (9,10). The lack of detailed information about biological parameters, however, restricts this purely theoretical approach to small circuit motifs with, at most, dozens of genes (11,12).

Single-cell data can potentially remedy the low dimensionality by providing high resolution on the expression patterns for tens of thousands of genes. Recently, several methods have been proposed to reconstruct an energy landscape using single-cell RNA sequencing (scRNA-seq) data using a variety of mathematical approaches including gene correlation, dynamical systems theory, and Fokker-Planck formalism (13–18). A key limitation of transcriptomics data is the challenge of extracting dynamical information about gene regulation and cell fate transitions from snapshot data that typically lack temporal information.

RNA splicing has been recently recognized as a promising avenue to study cell fate transitions and infer dynamical information from snapshot single-cell data (19,20). The underlying idea is that knowledge of the relative proportion of unspliced and spliced RNA counts can provide insight into gene expression dynamics due to the delay taken by RNA splicing (21). Specifically, RNA velocity employs a linear, mass action model based on ordinary differential equations that includes unspliced RNA production, splicing reaction generating spliced RNA, and spliced RNA degradation/dilution. By fitting this model with scRNA-seq data, RNA velocity successfully captures cell lineages in many biological contexts including pancreas development and neurogenesis (19,20). Recently, Dynamo provided a more general nonlinear framework for RNA splicing dynamics and even enabled the integration of traditional scRNA-seq with metabolic labeling (if available) for a more precise RNA velocity modeling by accurately estimating RNA-specific splicing rate constants (22). Moreover, we recently proposed spliceJAC, a multivariate model that captures RNA splicing as well as gene-gene interactions resulting in complex GRNs that are cell-type specific (23). Crucially, this model-based approach allowed us to estimate the stability of different cell types—identified in the model as the attractors in the complex gene-gene interaction landscape (24)—by reconstructing cell-type-specific Jacobian matrices. While these methods represent considerable steps forward in the integration of single cell data and rigorous modeling approaches, it remains challenging to characterize the tipping points—or transition points—that are responsible for cell fate decisions and potentially use this information to anticipate and predict critical transitions.

Here, we propose a new modeling strategy to analyze cell-state transition processes from single-cell transcriptomics data and identify unstable regions—or tipping points—in cell fate. Our method uses the unspliced and spliced scRNA-seq counts and lineage ordering to infer the stability of cell states and GRNs along the cell-state lineage. First, we demonstrate the ability of our method to correctly identify bifurcation structures in cell fate using synthetic data from small in silico circuits. Furthermore, we apply the method to two EMT time course data sets, identifying the stable epithelial and mesenchymal states as well the intermediate, unstable state. We further show that these tipping points are associated with rearrangement of the underlying gene circuitry, indicated, for example, by the increased number of connections between genes, that is reminiscent of critical transitions in classical physical systems. Overall, our model characterizes the cell-state trajectory during EMT and provides a new framework to identify transition states from single-cell transcriptomics data.

MATERIALS AND METHODS

We use a combination of dynamical system theory and machine-learning-oriented tools to identify and characterize transition states during cell-state transitions from single-cell transcriptomics data.

Model input

First, our computational pipeline takes as input the preprocessed RNA counts from scRNA-seq data (Fig. 1 *A*). Crucially, both the unspliced and spliced RNA counts are required to develop the RNA splicing model and downstream analysis, which could be extracted from raw scRNA-seq reads using several publicly available tools including velocyto and kallisto (20,25). Second, the method requires a lineage ordering of cells, with pseudotime being the overwhelmingly popular option (Fig. 1 *B*). Pseudotime metrics order cells along a "temporal" axis based on gene expression similarity, whereby cells in the starting state(s) (such as undifferentiated cells in stem cell development or epithelial cells in EMT) are expected to have low pseudotime values whereas cells in the terminal state(s) (such as differentiated cells or mesenchymal cells) are expected to have higher pseudotime values (26) (Fig. 1 *C*). Given the large number of available tools to estimate pseudotime, our pipeline allows flexibility to employ different pseudotime metrics including scanpy's diffusion pseudotime, scVelo's velocity pseudotime, and scFates (19,27,28).

Multivariate model of RNA splicing

We set up a multivariate model of RNA splicing based on our existing spliceJAC modeling framework (23), which is in turn an extension of the RNA velocity model (20). In our model, the dynamics of the unspliced (U_i) and spliced (S_i) copy number of a given RNA species (i) is governed by ordinary differentiation equations (ODE) as follows:

$$\frac{dU_i}{dt} = A\,S - \beta_i U_i \tag{1a}$$

$$\frac{dS_i}{dt} = \beta_i U_i - \gamma_i S_i \tag{1b}$$

where β_i is a splicing rate coefficient, γ_i is a degradation/dilution rate constant for spliced RNA species i, A is a cross-species interaction matrix, and $\boldsymbol{S} = (S_1, S_2, \ldots, S_N)$ is the vector of copy numbers for all RNA species. Therefore, A_{ij} represents how species j interacts with species i, whereby a positive, negative, or null coefficient represents transcriptional activation, inhibition, or lack thereof. Compared to the "standard" RNA velocity model that only features a constant production rate for unspliced RNA, Eqs. 1a and 1b introduce interactions between species and thus provide a strategy to infer the gene-gene interaction matrix A as discussed in the section below.

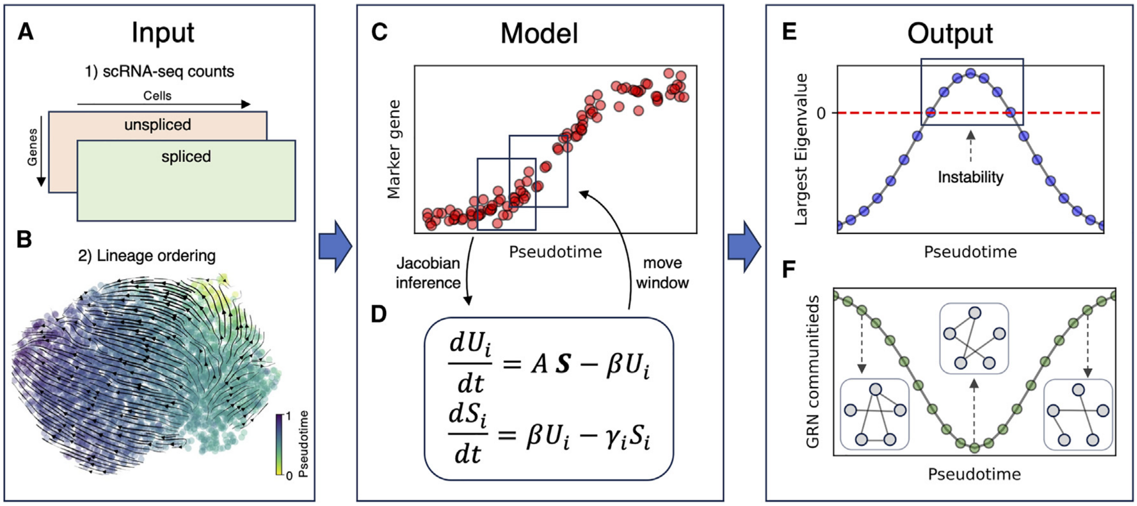

FIGURE 1 Workflow of computational analysis. The analysis requires (*A*) the unspliced and spliced counts from scRNA-seq data and (*B*) a lineage ordering of cells, such as pseudotime, overlayed here with RNA velocity transition trajectories. (*C*) Example of transition marker gene dynamics along pseudotime. After lineage sorting, an inference window is defined (*black box*). Cells within the window are used to construct a multivariate RNA splicing model (*D*), from which a Jacobian matrix is inferred. After each iteration, the inference window moves forward along the pseudotime axis. (*E*) Spectral analysis of the inferred Jacobian matrix identifies unstable points along the lineage based on positive eigenvalues. (*F*) A gene regulatory network (GRN) is constructed for each pseudotime point to inspect GRN rearrangement and organization through community detection.

Identification of tipping points along lineage

To infer information about stability and tipping points along the cell-state transition lineage, we set up an inference scheme based on the multivariate RNA splicing model (Fig. 1, *C* and *D*) and compute the Jacobian matrix associated with Eqs. 1a and 1b. First, cells are ordered along the chosen lineage-ordering metric (pseudotime in Fig. 1 *C*). Second, an "inference window" is defined to progressively scan the lineage. At each iteration, cells within the window are used to learn the parameters of the multivariate RNA splicing model of Eqs. 1a and 1b. At each "timestep," the Jacobian is composed by four quadrants corresponding to regulations between the unspliced and spliced RNA species (supporting methods 1). The cross-species interaction parameters for RNA species i are obtained by setting Eq. 1a to zero and solving the following regression problem:

$$A_{i0}^{*}, \left\{A_{ij}^{*}\right\} = \min_{A_{i0}, \left\{A_{ij}\right\}} \sum_{c} \left(A_{i0} + \sum_{j \neq i} A_{ij} S_j^{c} - \beta_i U_i^{c}\right)^2 + \lambda F\left(A_{i0}, \left\{A_{ij}\right\}\right), \tag{2}$$

where the subscript c denotes cells within the inference window. The last term in Eq. 2 is an additional constraint to the regression problem, for example in the case of lasso or ridge regression, where the shrinkage parameter λ can be set to a user-defined value. In the simpler case of linear regression, $\lambda = 0$. To further simplify the problem, we assume that the splicing rate coefficient is same for all RNA species. This assumption enables to set $\beta_i = \beta = 1$ by rescaling time in units of $1/\beta$ in Eqs. 1a and 1b. Finally, the species-dependent degradation/dilution rate coefficient γ_i can be inferred with linear regression after setting Eq. 1b to zero, $\gamma_i = U_i / S_i$.

By repeating these inference steps for each species, $i = (1, 2, \ldots M)$, the entire interaction matrix and degradation coefficients are obtained, thus allowing us to compute the Jacobian matrix of Eqs. 1a and 1b within the inference window. Afterward, the inference window is moved forward along the pseudotime axis until the entire lineage is analyzed.

The spectral analysis of the Jacobian matrix along the lineage is used to infer information about stability, for example by evaluating whether the largest eigenvector becomes positive, thus indicating instability (Fig. 1 *E*). The 1) width of the inference window, 2) increment between iterations, and 3) number of genes used in the model are free model parameters that can be modified by the user.

A key assumption in the inference scheme is the ordering of cells along a transition coordinate such as pseudotime, as temporal information is normally not available in single-cell transcriptomics data. Specifically, in the context of EMT, cells tend to undergo the transition in an asynchronous manner, thus leading to heterogeneous populations of cells with different epithelial-mesenchymal traits and rendering real-time information less effective (29).

GRN reconstruction and downstream analysis

The cross-species interaction matrix A inferred following the scheme of Eq. 2 can be interpreted as a GRN, which is used to study the rearrangement in gene regulation during the transition. To quantify the global change in the GRN along the lineage, we quantify the number of communities in the GRN graph defined as the highly connected sets of nodes (i.e., genes). First, we rescale the edges in the GRN based on the gene expression of the "sender" node (i.e., the regulator species). Therefore, the connection between genes is not activated if the regulator gene is not expressed. To estimate the communities, we employ different community search algorithms including the Girvan-Newman algorithm (30) and the Clauset-Newman-Moore greedy modularity maximization (31), both of which are implemented in the NetworkX python package. The number of communities is visualized along the lineage axis to showcase the emerging GRN structure (Fig. 1 *F*).

RESULTS

Identification of tipping points from in silico simulation

To test the method's ability to recover tipping points in cell-state transition, we first consider data generated in silico from stochastic simulations where a ground truth can be defined for benchmarking.

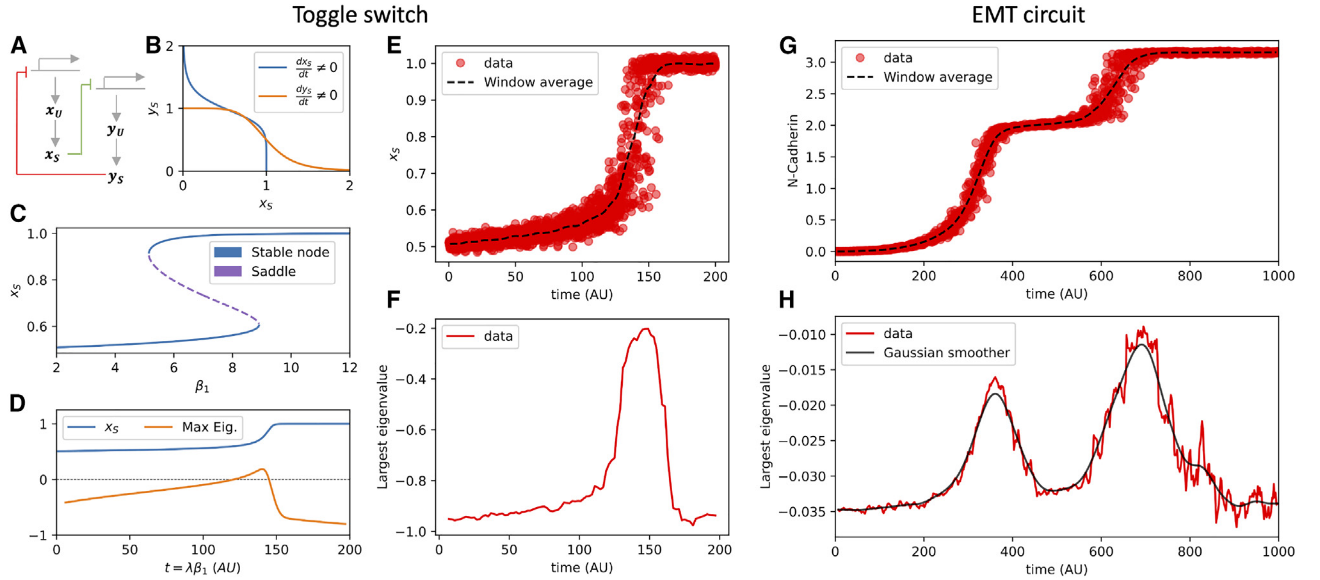

FIGURE 2 Detection of tipping points from simulation of circuit motifs. (*A*) The toggle switch motif including unspliced RNA production, splicing, degradation/dilution, and mutual feedback inhibition. (*B*) Phase diagram and nullclines of the toggle switch in the bistable regime. (*C*) Saddle-node bifurcation driven by increase of the parameter β_1. (*D*) Temporal dynamics of spliced X (x_S, *blue*) and largest Jacobian eigenvalue (*orange*) in a deterministic simulation as β_1 is slowly increased. Dashed black line indicates that the largest eigenvalue becomes positive at the bifurcating point. (*E*) Data points (*red*) and average trajectory (*black*) from stochastic simulation of the toggle switch. (*F*) Largest eigenvalue of the Jacobian matrix inferred from the simulated data points. (*G*) Data points (*red*) and average trajectory (*black*) from stochastic simulation of the tristable EMT circuit. (*H*) Largest eigenvalue of the Jacobian matrix inferred from the data points (*red*) of the EMT circuit and smoothened trajectory obtained via Gaussian filter (*black*). Parameter values for toggle switch and EMT simulation can be found in the supporting methods 2.1–2.2.

First, we simulate small, multistable circuits including a bistable toggle switch and a tristable circuit describing EMT using stochastic differential equations. The synthetic toggle switch is a simple motif composed by two genes (X and Y) that mutually repress each other, which can give rise to bistability between opposite cell states (32,33) (Fig. 2, *A* and *B*). To simulate the RNA splicing dynamics in the toggle switch, we generalize existing models to incorporate unspliced and spliced RNAs, resulting in four variables, including the unspliced and spliced counts for both X and Y (supporting methods 2.1). The circuit undergoes a saddle-node transition from a state with low expression of X to a state with high expression of X triggered by the increase of the feedback inhibition parameter β_1 (Fig. 2 *C*). To study the circuit's stability during the transition, we first compute the Jacobian matrix along a deterministic trajectory (i.e., ODE simulated without noise). As expected, the largest eigenvalue of the Jacobian matrix is negative toward the starting and terminal points of the trajectory, indicating stable fixed points, while it becomes positive in the intermediate region, thus indicating the transition through an unstable fixed point (Fig. 2 *D*). To define a more realistic biological scenario, we sample in silico data during the transition using stochastic ODE simulations and obtain a comprehensive representation of the initial state, transition cells, and terminal state (Fig. 2 *E*). When applying our Jacobian inference strategy to the sampled cells ordered by simulation time, we correctly observed a spike in the value of the Jacobian's largest eigenvalues corresponding to the saddle-node transition, demonstrating our method's ability to identify the saddle-node transition (Fig. 2 *F*). We further test how the Jacobian inference depends on the model parameters. The largest Jacobian eigenvalue approaches zero when the sample size of simulated data is large. Conversely, it spikes but remains negative when the sample size is smaller (Fig. S1 *A*). Furthermore, the width of Jacobian inference window and increment step influence the sharpness and location of the tipping point (Fig. S1, *B–D*). Moreover, we test how the timescales for parameter variation and cell-state transition interact. Specifically, cells do not complete the transition when the bifurcation parameter β_1 is increased quickly, thus not allowing cells enough time to complete the saddle-node transition. Conversely, longer simulation times enable a complete transition (Fig. S1, *E* and *F*).

Furthermore, we simulate a tristable GRN including epithelial noncoding RNAs and mesenchymal transcription factors that captures the EMT through an intermediate epithelial/mesenchymal state (5,34) (supporting methods 2.2). Therefore, a complete EMT trajectory is characterized by two successive saddle-node transitions including epithelial to intermediate and intermediate to mesenchymal, which are captured by stochastic simulations of the circuit (Fig. 2 *G*). Consistently, our method captures the tipping points separating the epithelial, intermediate and mesenchymal stable cell states as spikes in the value of the largest

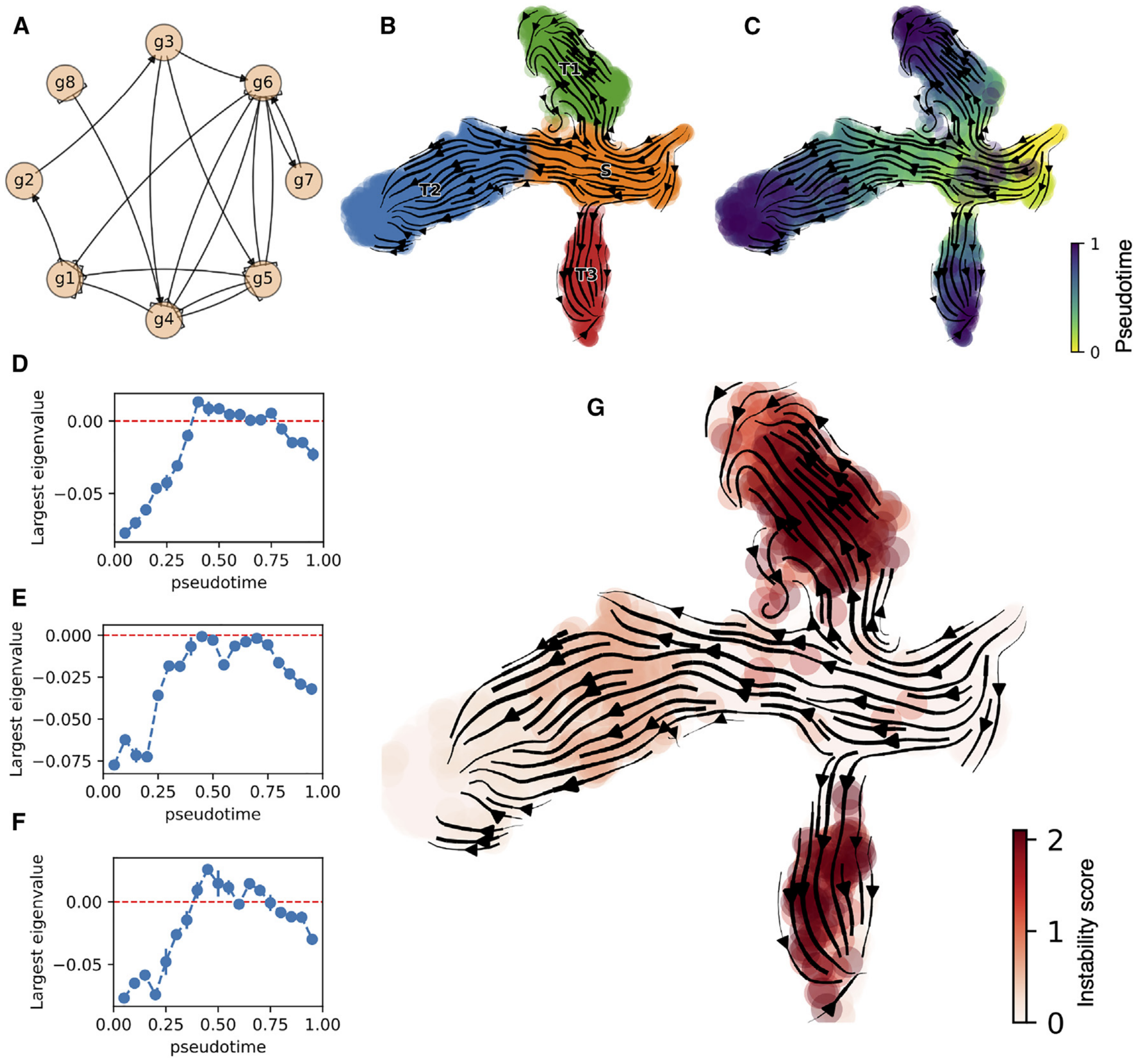

FIGURE 3 Inference of tipping points from in silico scRNA-seq data of trifurcating circuit. (*A*) The trifurcating circuit schematic. (*B*) Low-dimensional uniform manifold approximation and projection (UMAP) embedding, clustering, and RNA velocity highlighting the cell-state transitions. (*C*) Simulation pseudotime computed via BoolODE. (*D–F*) Largest eigenvalue of the Jacobian matrix for the three cell-state transition trajectories S-T1 (*D*), S-T2 (*E*), and S-T3 (*F*). The dashed horizontal line highlights positive values. (*G*) Instability score in low-dimensional UMAP embedding highlights the tipping points of the trifurcating circuit.

Jacobian eigenvalue inferred from the simulated data (Fig. 2 *H*). While the overall trend of the largest Jacobian eigenvalue suggests subsequent saddle-node bifurcations, the trajectory exhibits stochastic fluctuations that arise as high-frequency variation (see Fig. 2 *H*, *red curve*). These fluctuations are attenuated by increasing the sampling size of simulated cells while increasing the noise amplitude in the simulated data (Fig. S2).

Next, to test the method in a more complex scenario where multiple choices of cell fate are available in the cell lineage, we consider a trifurcating circuit whereby cells in the starting state (S) can differentiate into one of three final cell states (T1, T2, T3) (Fig. 3 *A*). We generated an in silico scRNA-seq data set by simulating the dynamics of the trifurcating circuit using the BoolODE package (35) (supporting methods 2.3), whereby cells start in the initial state and differentiate through one of the three branches during the simulation, which can be visualized in low-dimensional embedding of the data (Fig. 3, *B* and *C*). First, we focused on the individual "branches" of the lineage, where different genes are selectively turned on or off in a branch-specific manner (Fig. S3). To study the stability along individual branches, we select only cells in the starting and final state of choice and apply our stability analysis. In all three branches, our method correctly identifies the stable attractors corresponding to initial (S) and final (T1, T2, or T3) states based on negative eigenvalues of the inferred Jacobian matrix. Moreover, the largest eigenvalue of the inferred Jacobian matrix transiently becomes positive in the region separating the stable states in all three branches, indicating

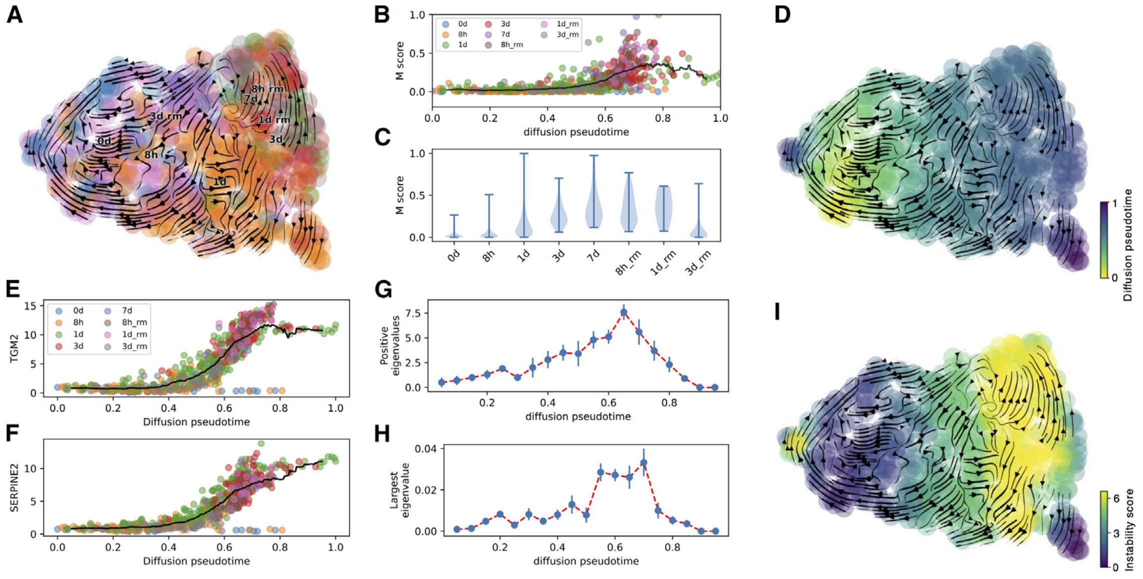

FIGURE 4 Saddle-node bifurcation and tipping point during EMT in OVCA420 cells. (*A*) Low-dimensional UMAP embedding and time course labels of the OVCA420 time course data set. (*B*) Mesenchymal score of individual cells as a function of diffusion pseudotime. (*C*) Violin plot of mesenchymal score per time point. Error bars showcase extremal values. (*D*) Low-dimensional UMAP embedding and diffusion pseudotime. (*E* and *F*) Expression of EMT response genes as a function of pseudotime. (*G* and *H*) Number of positive Jacobian eigenvalues (*G*) and largest Jacobian eigenvalue (*H*) as a function of diffusion pseudotime. Error bars showcase standard deviation over $n = 10$ Jacobian inference iterations. (*I*) Low-dimensional UMAP embedding and instability score. In (*B*), (*E*), and (*F*), colors indicate experimental time marks, while the black line showcases the trajectory average.

the unstable region of transition (Fig. 3, *D–F*). Finally, by integrating the instability scores along individual branches (supporting methods 3), we defined a global instability score that highlights the attractor basins in the landscape (S, T1, T2, T3) and the transition regions or tipping points separating them (Fig. 3 *G*).

Overall, the benchmarking on synthetic data acquired both via custom-made stochastic simulations and external tools supports our method's ability to identify the stable attractors and tipping points during cell-state transitions. Next, we apply the methodology to real data sets to characterize transitions and gene regulation during EMT.

Detection of saddle-node transition during EMT

Next, we test the method on real biological data. First, we consider a scRNA-seq time course data set of ovarian cancer epithelial cells OVCA420 undergoing EMT (36). In the experimental setup, epithelial cancer cells were exposed to transforming growth factor β1—a well-known EMT inducer—for a week, followed by a remission period of 3 days without any external stimulus. scRNA-seq was performed at multiple time points, leading to an aggregated data set with cells with different epithelial-mesenchymal traits (Fig. 4 *A*). The transition toward a mesenchymal state was further highlighted by the increase of a mesenchymal score, defined as the average expression of a mesenchymal gene signature (37–39) (Fig. 4, *B* and *C*). Pseudotime and RNA velocity analysis of the data set further suggest that epithelial cells, mostly identified at early time points in the experiment, gradually transition and become mesenchymal cells mostly found at later time points (Fig. 4 *D*).

Next, we apply our inference scheme to inspect the stability along the EMT trajectory. We use diffusion pseudotime to order cells along an EMT trajectory. This choice is supported by the observed dynamics of known EMT marker genes such as TGM2 and SERPINE2, which exhibit low expression toward the beginning of the lineage and are activated at later stages (Fig. 4, *E* and *F*). When applying our Jacobian inference method on the EMT lineage, we inferred the typical saddle-node transition previously observed in synthetic circuits characterized by two stable states at the beginning and end of the trajectory separated by a more unstable region. Notably, the unstable region emerged when inspecting both the value of the largest Jacobian eigenvalue as well as the overall number of positive eigenvalues (Fig. 4, *G* and *H*). Interestingly, a small number of Jacobian eigenvalues remain positive even in the sections of the lineage corresponding to the epithelial and mesenchymal attractors. To further investigate this finding, we evaluate the number of positive Jacobian eigenvalues along the EMT lineage when the dimensionality of the system is increased by selecting more top-expressed genes (see supporting methods 5). Interestingly, the system conserves the predicted saddle-node transition characterized by a peak in the number of positive eigenvalues when the dimensionality is increased, but the number of positive eigenvalues rises (Fig. S4). When normalizing the number of positive eigenvalues by the total number of genes, however, systems with different dimensionality behave similarly,

especially near the area instability (Fig. S4 *C*). To further test the robustness of the inferred tipping point, we perform extensive sensitivity analysis showing that the saddle-node lineage structure is conserved upon variation of the inference scheme parameters including the width and increment of the inference window (Fig. S5). The typical saddle-node behavior is further observed when a user-curated list of epithelial and mesenchymal genes is used as gene set for Jacobian inference (38), further confirming the robustness of the prediction (Fig. S6). Finally, the stability along the EMT trajectory is visualized in low-dimensional uniform manifold approximation and projection embedding, which highlights a stable epithelial state, an intermediate and unstable region corresponding to transitioning cells, and a stable mesenchymal state that acts as a convergence point for RNA velocity transition trajectories (Fig. 4 *J*).

Moreover, we compare our calculation of the state-dependent Jacobian matrix with Dynamo, a model based on RNA splicing dynamics that reconstructs vector fields from scRNA-seq data (22). To ensure a common ground for the comparison, we average the Jacobian matrix predicted by Dynamo over the same diffusion pseudotime window (supporting methods 7) and compare the predicted stability of the EMT trajectory. We compare the methods under different conditions for Jacobian inference, including by 1) letting Dynamo's preprocessing pick the top candidate genes and 2) manually enforcing the same gene set used for our calculation. We find that, irrespective of gene set choice, our method uniquely predicts the instability associated with the EMT saddle-node transition, whereas Dynamo consistently predicts instability throughout the lineage (Fig. S7). It is worth noting that this comparison does not imply a general trend, and a more thorough evaluation across different biological systems with various degrees of complexity would be necessary to extrapolate general conclusions. For example, Dynamo reconstructs genome-wide, rather than local, vector fields, thus potentially hampering its ability to capture the local behavior in specific circumstances, for example when the sample size in the data set is small, possibly explaining the better performance of our linear model.

Finally, we test how real-time information could inform the stability analysis of the OVCA420 EMT lineage by inferring the Jacobian matrix over the time points of the time course, which include five time points for EMT induction and three time points for remission. Interestingly, the largest Jacobian eigenvalue peaks 3 days after the beginning of EMT induction, thus resembling the characteristic saddle-node behavior observed along the pseudotime axis. The number of positive eigenvalues, however, fluctuates and does not show a consistent trend (Fig. S8). Overall, these observations suggest that pseudotime might be a better "reaction coordinate" to describe EMT in this particular data set.

To test the saddle-node bifurcation structure inferred in the OVCA420 EMT trajectory, we repeated the analysis on a different cell line generated in the same original experiment. Specifically, we considered the response of the A549 cell line derived from lung cancer when exposed to the same EMT inducer (transforming growth factor β1) (Fig. 5 *A*). Notably, the diffusion pseudotime used as the lineage-ordering parameter for OVCA420 cells failed to correlate with experimental time for the A549 data set (Fig. S9, *A–C*). For this reason, we instead considered velocity pseudotime as an alternative ordering parameter. While diffusion pseudotime is implemented in the scanpy's package and solely relies on gene expression cell-cell similarity, velocity pseudotime is implemented in the scVelo package and also considers RNA velocity trajectories to rank cells. When using velocity pseudotime as lineage-ordering parameter, A549 cells consistently showed a transition toward a mesenchymal state highlighted by an increase in the mesenchymal score (Fig. 5, *B* and *C*). Crucially, the Jacobian inference along the velocity pseudotime axis confirmed a saddle-node-like bifurcation structure with spikes in both the largest Jacobian eigenvalue and the overall number of positive eigenvalues (Fig. 5, *D* and *E*), which was further robust upon variation of inference parameters (Fig. S9, *D–G*). When visualized on uniform manifold approximation and projection embedding, the resulting instability score highlighted the starting and terminal states separated by an instability region (Fig. 5 *F*).

Emergent rearrangement of GRNs during EMT

Finally, it is expected that cell transitions result in a rearrangement of gene-gene interactions exemplified by changes in the GRN. Starting from the inferred Jacobian matrix, a GRN can be reconstructed to capture the key interactions between genes at each point in the cell lineage (supporting methods 1), resulting in a description of the rearrangement of gene-gene interactions during EMT (Fig. 6, *A–G*). From the inferred GRN, it is further possible to identify the key interactions characterizing the cellular states in the epithelial and mesenchymal attractors as well as the intermediate tipping point (Fig. S10).

Furthermore, we apply community detection algorithms to the inferred GRN graph to summarize the emerging GRN evolution along the EMT lineage at a more coarse-grained level. Specifically, we apply community detection algorithms including the Girvan-Newman and greedy modularity algorithms (supporting methods 8) to quantify the changing organization of the GRN during EMT. When applied to the OVCA420 and A549 cancer cell line data sets, both algorithms detect a large number of GRN communities at the beginning of the lineage corresponding to the initial, epithelial state. The number of communities, however, decreases significantly in the tipping point region corresponding to the intermediate, unstable state before increasing again toward the terminal, mesenchymal state (Fig. 6, *H–J*). This result suggests that interactions between

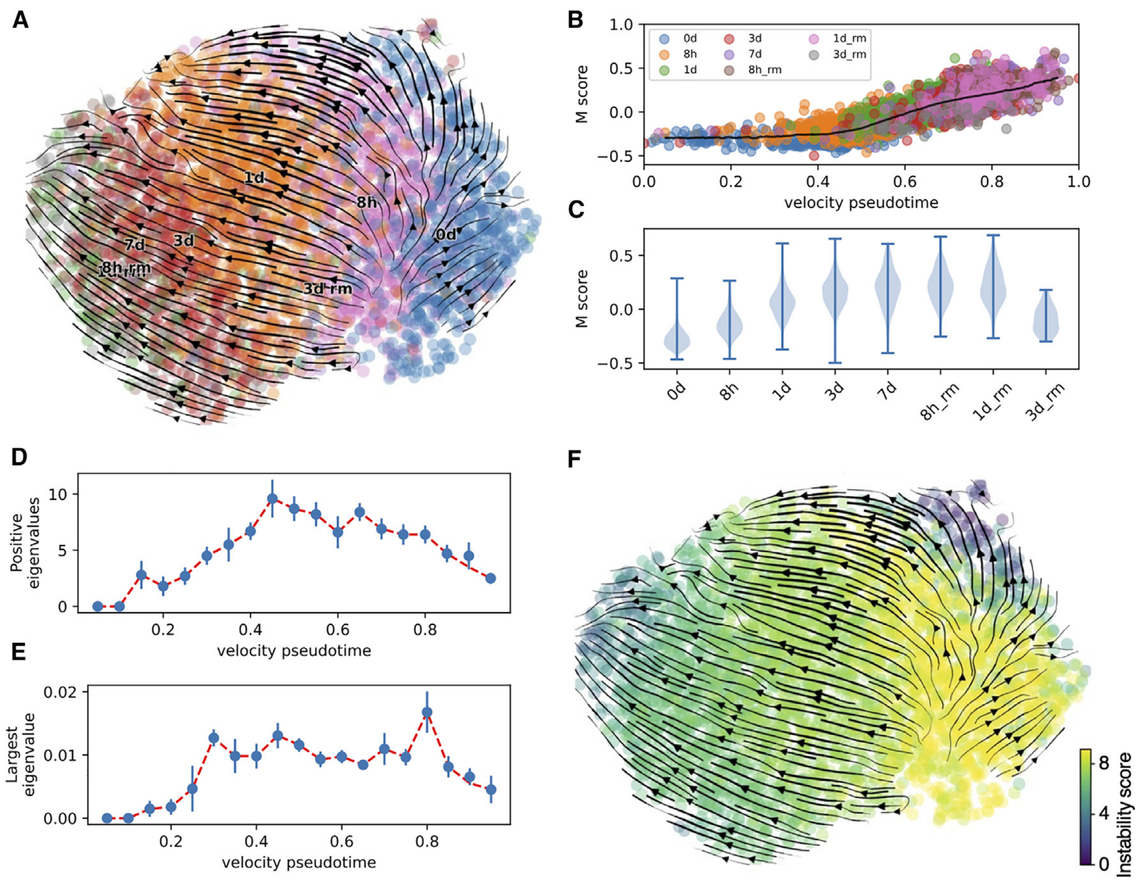

FIGURE 5 Saddle-node bifurcation and tipping point during EMT in A549 cells. (*A*) Low-dimensional UMAP embedding and time course labels of the OVCA420 time course data set. (*B*) Mesenchymal score of individual cells as a function of diffusion pseudotime. (*C*) Violin plot of mesenchymal score per time point. Error bars showcase extremal values. (*D* and *E*) Number of positive Jacobian eigenvalues (*D*) and largest Jacobian eigenvalue (*E*) as a function of diffusion pseudotime. Error bars showcase standard deviation over n = 10 Jacobian inference iterations. (*F*) Low-dimensional UMAP embedding and instability score. In (*B*), colors indicate experimental time marks, while the black line showcases the trajectory average.

genes are more compartmentalized in the epithelial and mesenchymal states, with GRNs exhibiting a modular structure. Conversely, genes are more highly connected in the intermediate, unstable state. This observation is supported by inspecting the overall GRN edge weight distribution at different points in pseudotime. The edge weight distribution is narrower for pseudotime points corresponding to the stable E and M states—indicting that fewer gene-gene connections are significant—while being broader for intermediate pseudotime points corresponding to the tipping point—indicating a larger number of connections between genes (Fig. 6 *K*).

Overall, these results suggest a rearrangement of the connections in the GRN during the EMT trajectory.

DISCUSSION

Learning dynamical information from static single-cell transcriptomics data is a key challenge where mathematical and computational modeling can provide transparent and falsifiable predictions. Here, we have presented a new method that utilized the RNA counts from scRNA-seq data to build a multivariate model of RNA splicing and the tipping points in cell fate along cell lineages. Using this method, we identified the intermediate, unstable cell states during EMT and further characterized the GRN associated with the transition.

Previously, we adopted a steady-state assumption to solve an interacting RNA splicing model and characterize gene expression within individual cell states (23). Here, we have extended this framework to intermediate cell states along the epithelial-mesenchymal lineage. This strategy is justifiable in the context of EMT when considering that the timescales of RNA splicing and EMT are well separated. While RNA splicing reactions have a typical timescale of ∼10 min, EMT progression typically requires up to 3–5 days (2,21). Furthermore, the epithelial-mesenchymal spectrum is characterized by intermediate checkpoints whereby hybrid epithelial/mesenchymal cell states can be maintained over many cell division cycles (2). These specific considerations might explain why our approach recapitulates the saddle-node transition in the EMT trajectory

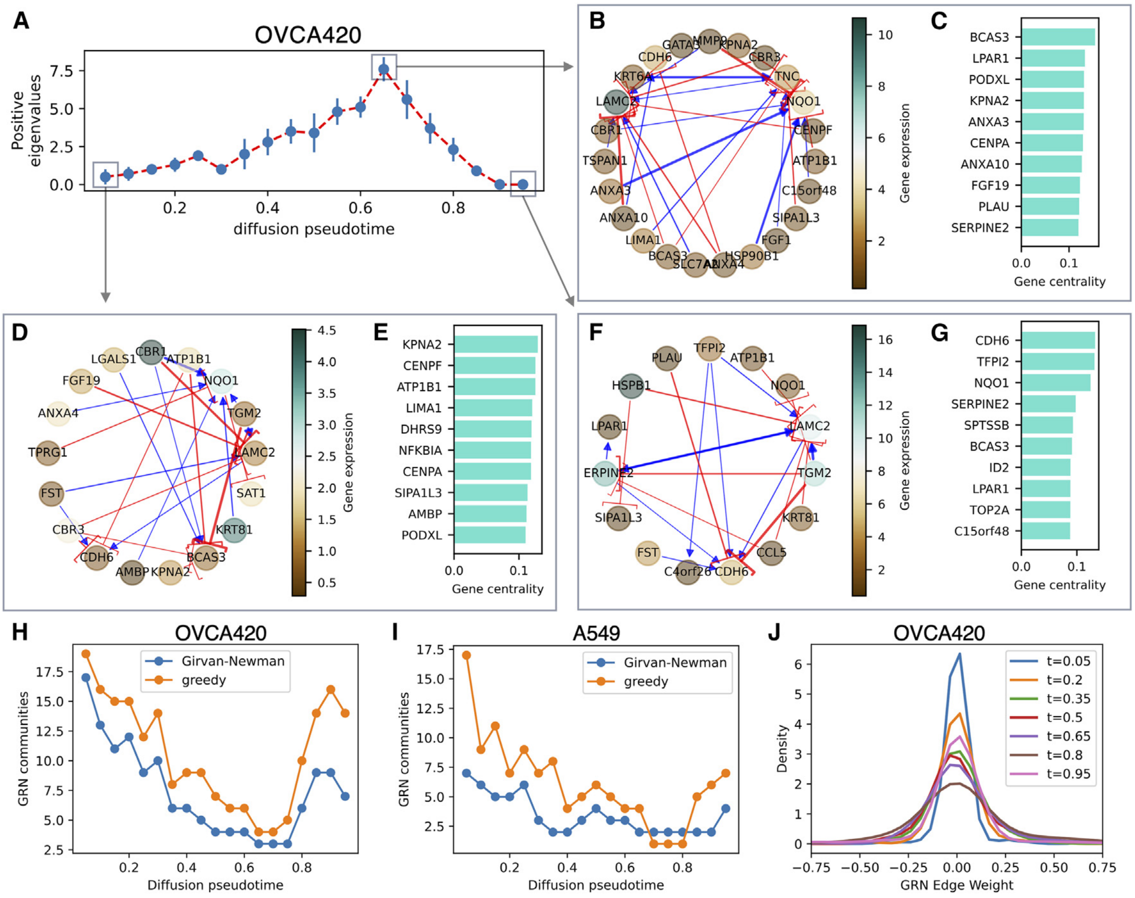

FIGURE 6 Variation of gene regulatory network during EMT. (*A*) The saddle-node behavior exemplified by the number of positive eigenvalues as a function of diffusion pseudotime in the OVCA420 cell line. Error bars showcase standard deviation over $n = 10$ Jacobian inference iterations. (*B* and *C*) Core gene regulatory network and top genes ranked by betweenness centrality in the tipping point of the OVCA420 lineage. Blue and red arrows indicate activation and inhibition, respectively, while the node colormap indicated gene expression. (*D* and *E*) Same as (*B*) and (*C*) in the pseudotime point corresponding to the epithelial attractor. (*F* and *G*) Same as (*B*) and (*C*) in the pseudotime point corresponding to the mesenchymal attractor. (*H*) Number of communities in the GRN graph as a function of diffusion pseudotime in the EMT trajectory of OVCA420 cells under TGFB1 induction. (*I*) Same as (*H*) for the A549 cell line data set. (*J*) The GRN edge weight distribution in the OVCA420 data set GRN for different points in pseudotime.

whereas nonequilibrium methods such as Dynamo consistently predict instability throughout the EMT lineage (see Fig. S7). Specifically, Dynamo does not assume equilibration of the RNA splicing reaction but rather uses RNA velocity to solve a time-dependent, nonequilibrated process, potentially explaining the different prediction (22). Certainly, our approach could be improved in the future by combining interacting RNA splicing models with more sophisticated inference schemes that do not require steady-state assumptions, such as Dynamo.

Compared to existing methods relying on single-cell transcriptomics data, our model presents significant advantages. First, several methods reconstruct the underlying gene expression landscape by relying exclusively on standard scRNA-seq data (i.e., only the spliced RNA counts). For example, MuTrans employs a multiscale reduction technique and constructs a dynamical manifold based on Langevin equation and rate theory that depicts stable and transitioning cells as well as transition paths (18). Moreover, GraphFP (16) employs the Fokker-Planck formalism to construct a transition graph between cells using time series transcriptomics data. Conversely, scEpath constructs a landscape based on single-cell energy whereby the energy is defined based on maximum entropy of a gene-gene correlation graph within individual cells (15). DensityPath constructs an intrinsic landscape structure in the low-dimensional embedding of the data on which optimal transition paths are inferred (14). The landscape of differentiation dynamics models a stochastic process based on the Fokker-Planck equation to learn cell population density and reconstruct a lineage in pseudotime (13). Compared to these methods, our model has the distinct advantage of utilizing both the unspliced and spliced RNA counts from single-cell transcriptomics, which provides more information about gene regulation and enables us to infer a directed GRN and Jacobian matrix to quantify cell-state stability.

Furthermore, a few existing methods include unspliced RNA counts for better modeling and inference. First, RNA

velocity builds an ordinary differential equation-based model including RNA production, splicing, and degradation (19,20). Recently, Dynamo presented a framework to reconstruct analytical vector fields and learn systems biology models from RNA velocity vectors by assuming Markovian dynamics. In this sense, our proposed method can be understood as a local linear approximation of the more general nonlinear vector field predicted by Dynamo (22). Recently, this framework was extended to preserve velocity magnitude in low-dimensional embedding and reconstruct a data-driven Fokker-Planck equation that captures the transition dynamics in the entire data set (40). Similarly, scMomentum uses RNA velocity to infer a gene-gene interaction matrix and further defines an energy landscape based on a Hopfield model using gene-gene interactions (41). Compared to these methods, which fit noninteracting models to the data, we develop a multivariate model including transcriptional regulation between genes. Therefore, we are able to infer a Jacobian matrix that encodes information about cell-state stability and GRNs. Recently, Wang and collaborators proposed an interacting RNA splicing model to quantify the GRN along a cell phenotype transition reaction coordinate (42). In their framework, the transition path between two cell-state attractors is identified using a finite temperature string method in conjunction with a nonlinear vector field computed with Dynamo. Notably, when applied to the A549 single-cell time course EMT data set, the authors reached a similar conclusion that the gene-gene interaction density—quantified in their work in terms of the GRN frustration—is maximal in the intermediate, transitory state, reflecting our finding that the number of communities in the GRN graph is minimized in the intermediate, transitory state.

CONCLUSIONS

While providing new, exciting insight, we acknowledge existing limitations in our model. One potential drawback of our approach is the reliance on lineage inference methods, such as pseudotime, to order cells along transition trajectories. Specifically, pseudotime might provide conflicting predictions on cell ordering, especially in more complex lineage structures including multiple coexisting cell states and choices in cell fate commitment. Extending the existing inference framework to such complex lineage structures could be the focus of future research, perhaps by integrating our model with existing tools for to infer lineage structure and/or by exploring data sets with real-time information such as metabolic label-based transcriptomics. Another important limitation of our approach is a constant splicing rate for all RNA species. This assumption, which is shared with several existing RNA splicing models including velocyto, scVelo, and spliceJAC (43), is not necessarily biologically accurate and might be revisited in the presence of additional information to estimate gene-dependent splicing rates, such as the above-mentioned metabolic labeling-based scRNA-seq, following a modeling strategy similar to Dynamo (22). Furthermore, it is assumed in our model of gene-gene interactions that the RNA abundance could approximate the copy number of transcription factors well, which might not necessarily be true. An interesting future direction would be the integration of single-cell transcriptomics data with protein-level measurements, such as protein fluorescence or the promising single-cell proteomics. When combined with transcriptomics data, protein data would provide more insight into gene expression dynamics and could potentially help to benchmark the prediction of GRN. Furthermore, data sparsity represents a key drawback of scRNA-seq, which can potentially compromise the quality of the inference. Including both unspliced and spliced RNA counts potentially amplifies the sparsity problem, as unspliced RNAs are typically rarer than spliced RNAs (about 10%–20% of the total RNA population). Tackling these existing issues with more elaborate models might, in the future, provide an even more accurate picture of the EMT process and reconcile observations that are currently poorly understood, such as our model's lack of a true steady state exemplified by positive Jacobian eigenvalue in the epithelial and mesenchymal cell-state attractors. Moreover, it will enable the application of this model to nonequilibrium biological processes whereby steady-state assumptions might not be feasible.

SUPPORTING MATERIAL

Supporting material can be found online at https://doi.org/10.1016/j.bpj.2024.03.021.

AUTHOR CONTRIBUTIONS

M.B. and F.B. performed the research; F.B. and Q.N. conceptualized the research; F.B. wrote the manuscript with input from all authors; and Q.N. supervised the research and acquired funding.

ACKNOWLEDGMENTS

The work was supported by National Science Foundation grants DMS1763272 and MCB2028424, a grant from the Simons Foundation (594598 to Q.N.), and National Institutes of Health grants R01AR079150 and U01AR073159.

DECLARATION OF INTERESTS

The authors declare no competing interests.

REFERENCES

1. Dongre, A., and R. A. Weinberg. 2019. New insights into the mechanisms of epithelial-mesenchymal transition and implications for cancer. *Nat. Rev. Mol. Cell Biol.* 20:69–84.

2. Jia, D., X. Li, …, H. Levine. 2019. Quantifying Cancer Epithelial-Mesenchymal Plasticity and its Association with Stemness and Immune Response. *J. Clin. Med.* 8, 725.
3. Vegliante, R., I. Pastushenko, and C. Blanpain. 2022. Deciphering functional tumor states at single-cell resolution. *EMBO J.* 41, e109221.
4. Lu, M., M. K. Jolly, …, E. Ben-Jacob. 2013. MicroRNA-based regulation of epithelial-hybrid-mesenchymal fate determination. *Proc. Natl. Acad. Sci. USA.* 110:18144–18149.
5. Tian, X. J., H. Zhang, and J. Xing. 2013. Coupled reversible and irreversible bistable switches underlying TGFβ-induced epithelial to mesenchymal transition. *Biophys. J.* 105:1079–1089.
6. Ramirez, D., V. Kohar, and M. Lu. 2020. Toward Modeling Context-Specific EMT Regulatory Networks Using Temporal Single Cell RNA-Seq Data. *Front. Mol. Biosci.* 7:54.
7. Waddington, C. H. 1957. The Strategy of the Genes.
8. Ferrell, J. E. 2012. Bistability, bifurcations, and Waddington's epigenetic landscape. *Curr. Biol.* 22:R458–R466.
9. Wang, J., K. Zhang, …, E. Wang. 2011. Quantifying the Waddington landscape and biological paths for development and differentiation. *Proc. Natl. Acad. Sci. USA.* 108:8257–8262.
10. Li, C., T. Hong, and Q. Nie. 2016. Quantifying the landscape and kinetic paths for epithelial-mesenchymal transition from a core circuit. *Phys. Chem. Chem. Phys.* 18:17949–17956.
11. Wang, J. 2015. Landscape and flux theory of non-equilibrium dynamical systems with application to biology. *Adv. Phys.* 64:1–137.
12. Bocci, F., D. Jia, …, J. Onuchic. 2023. Theoretical and computational tools to model multistable gene regulatory networks. *Rep. Prog. Phys.* 86, 106601.
13. Shi, J., T. Li, …, K. Aihara. 2019. Quantifying pluripotency landscape of cell differentiation from scRNA-seq data by continuous birth-death process. *PLoS Comput. Biol.* 15, e1007488.
14. Chen, Z., S. An, …, L. Wan. 2019. DensityPath: an algorithm to visualize and reconstruct cell state-transition path on density landscape for single-cell RNA sequencing data. *Bioinformatics.* 35:2593–2601.
15. Jin, S., A. L. MacLean, …, Q. Nie. 2018. scEpath: energy landscape-based inference of transition probabilities and cellular trajectories from single-cell transcriptomic data. *Bioinformatics.* 34:2077–2086.
16. Jiang, Q., S. Zhang, and L. Wan. 2022. Dynamic inference of cell developmental complex energy landscape from time series single-cell transcriptomic data. *PLoS Comput. Biol.* 18, e1009821.
17. Schiebinger, G., J. Shu, …, E. S. Lander. 2019. Optimal-Transport Analysis of Single-Cell Gene Expression Identifies Developmental Trajectories in Reprogramming. *Cell.* 176:1517–1943.e22.
18. Zhou, P., S. Wang, …, Q. Nie. 2021. Dissecting transition cells from single-cell transcriptome data through multiscale stochastic dynamics. *Nat. Commun.* 12:5609.
19. Bergen, V., M. Lange, …, F. J. Theis. 2020. Generalizing RNA velocity to transient cell states through dynamical modeling. *Nat. Biotechnol.* 38:1408–1414.
20. La Manno, G., R. Soldatov, …, P. V. Kharchenko. 2018. RNA velocity of single cells. *Nature.* 560:494–498.
21. Shamir, M., Y. Bar-On, …, R. Milo. 2016. SnapShot: Timescales in Cell Biology. *Cell.* 164:1302–1302.e1.
22. Qiu, X., Y. Zhang, …, J. S. Weissman. 2022. Mapping transcriptomic vector fields of single cells. *Cell.* 185:690–711.e45.
23. Bocci, F., P. Zhou, and Q. Nie. 2022. spliceJAC: transition genes and state-specific gene regulation from single-cell transcriptome data. *Mol. Syst. Biol.* 18, e11176.
24. Huang, S., G. Eichler, …, D. E. Ingber. 2005. Cell fates as high-dimensional attractor states of a complex gene regulatory network. *Phys. Rev. Lett.* 94, 128701.
25. Melsted, P., A. S. Booeshaghi, …, L. Pachter. 2021. Modular, efficient and constant-memory single-cell RNA-seq preprocessing. *Nat. Biotechnol.* 39:813–818.
26. Trapnell, C., D. Cacchiarelli, …, J. L. Rinn. 2014. The dynamics and regulators of cell fate decisions are revealed by pseudotemporal ordering of single cells. *Nat. Biotechnol.* 32:381–386.
27. Faure, L., R. Soldatov, …, I. Adameyko. 2023. scFates: a scalable python package for advanced pseudotime and bifurcation analysis from single-cell data. *Bioinformatics.* 39, btac746.
28. Wolf, F. A., P. Angerer, and F. J. Theis. 2018. SCANPY: large-scale single-cell gene expression data analysis. *Genome Biol.* 19:15.
29. Sha, Y., S. Wang, …, Q. Nie. 2021. Inference of Intercellular Communications and Multilayer Gene-Regulations of Epithelial–Mesenchymal Transition From Single-Cell Transcriptomic Data. *Front. Genet.* 11:11.
30. Girvan, M., and M. E. J. Newman. 2002. Community structure in social and biological networks. *Proc. Natl. Acad. Sci. USA.* 99:7821–7826.
31. Clauset, A., M. E. J. Newman, and C. Moore. 2004. Finding community structure in very large networks. *Phys. Rev. E.* 70, 066111.
32. Xu, L., K. Zhang, and J. Wang. 2014. Exploring the Mechanisms of Differentiation, Dedifferentiation, Reprogramming and Transdifferentiation. *PLoS One.* 9, e105216.
33. Verd, B., A. Crombach, and J. Jaeger. 2014. Classification of transient behaviours in a time-dependent toggle switch model. *BMC Syst. Biol.* 8:43.
34. Zhang, J., X. J. Tian, …, J. Xing. 2014. TGF-β-induced epithelial-to-mesenchymal transition proceeds through stepwise activation of multiple feedback loops. *Sci. Signal.* 7, ra91.
35. Pratapa, A., A. P. Jalihal, …, T. M. Murali. 2020. Benchmarking algorithms for gene regulatory network inference from single-cell transcriptomic data. *Nat. Methods.* 17:147–154.
36. Cook, D. P., and B. C. Vanderhyden. 2020. Context specificity of the EMT transcriptional response. *Nat. Commun.* 11:2142.
37. Chakraborty, P., J. T. George, …, M. K. Jolly. 2020. Comparative Study of Transcriptomics-Based Scoring Metrics for the Epithelial-Hybrid-Mesenchymal Spectrum. *Front. Bioeng. Biotechnol.* 8:220.
38. Foroutan, M., J. Cursons, …, M. J. Davis. 2017. A Transcriptional Program for Detecting TGFβ-Induced EMT in Cancer. *Mol. Cancer Res.* 15:619–631.
39. Bocci, F., P. Zhou, and Q. Nie. 2021. Single-Cell RNA-Seq Analysis Reveals the Acquisition of Cancer Stem Cell Traits and Increase of Cell-Cell Signaling during EMT Progression. *Cancers.* 13, 5726.
40. Zhang, Y., X. Qiu, …, J. Xing. 2023. Graph-Dynamo: Learning stochastic cellular state transition dynamics from single cell data. Preprint at bioRxiv. https://doi.org/10.1101/2023.09.24.559170.
41. Soto, L. M., J. P. Bernal-Tamayo…, 2020. scMomentum: Inference of Cell-Type-Specific Regulatory Networks and Energy Landscapes. Preprint at bioRxiv. https://doi.org/10.1101/2020.12.30.424887.
42. Wang, W., K. Ni, …, J. Xing. 2021. Transiently increased intercommunity regulation characterizes concerted cell phenotypic transition. Preprint at bioRxiv. https://doi.org/10.1101/2021.09.21.461257.
43. Xing, J. 2022. Reconstructing data-driven governing equations for cell phenotypic transitions: integration of data science and systems biology. *Phys. Biol.* 19, 061001.

Biophysical Journal, Volume *123*

Supplemental information

Tipping points in epithelial-mesenchymal lineages from single-cell transcriptomics data

Manuel Barcenas, Federico Bocci, and Qing Nie

Supplementary Material

Tipping points in epithelial-mesenchymal lineages from single cell transcriptomics data

Running title: detecting bifurcations in scRNA-seq data

Manuel Barcenas[1,*], Federico Bocci[1,2,*,#], Qing Nie[1,2,#]

[1]Department of Mathematics and [2]NSF-Simons Center for Multiscale Cell Fate Research, University of California Irvine, 92697 Irvine, CA, USA
*These authors equally contributed to the research

[#]Authors for correspondence:
Qing Nie: qnie@uci.edu
Federico Bocci: fbocci@uci.edu

Supplementary Methods

1. Jacobian of the interacting RNA splicing model

Starting from eqs. (1), we rewrite the interacting RNA splicing model in terms of the functions f_i, g_i defined as:

$$\frac{dU_i}{dt} = f_i(\boldsymbol{U}, \boldsymbol{S}) = \sum_{j=1}^{N} a_{ij} S_j - U_i \quad (1a)$$

$$\frac{dS_i}{dt} = g_i(\boldsymbol{U}, \boldsymbol{S}) = U_i - \gamma_i S_i \quad (1b)$$

Where we have assumed $\beta_i = \beta = 1$ as explained in the main text, N is number of interacting RNA species, and the coefficients a_{ij} quantify the regulation of species j in species i. The Jacobian matrix is defined as:

$$J = \begin{pmatrix} \frac{\partial f_1}{\partial U_1} & \cdots & \frac{\partial f_1}{\partial U_N} & \frac{\partial f_1}{\partial S_1} & \cdots & \frac{\partial f_1}{\partial S_N} \\ \vdots & & \vdots & \vdots & & \vdots \\ \frac{\partial f_N}{\partial U_1} & \cdots & \frac{\partial f_N}{\partial U_N} & \frac{\partial f_N}{\partial S_1} & \cdots & \frac{\partial f_N}{\partial S_N} \\ \frac{\partial g_1}{\partial U_1} & \cdots & \frac{\partial g_1}{\partial U_N} & \frac{\partial g_1}{\partial S_1} & \cdots & \frac{\partial g_1}{\partial S_N} \\ \vdots & & \vdots & \vdots & & \vdots \\ \frac{\partial g_N}{\partial U_1} & \cdots & \frac{\partial g_N}{\partial U_N} & \frac{\partial g_N}{\partial S_1} & \cdots & \frac{\partial g_N}{\partial S_N} \end{pmatrix} = \begin{pmatrix} -1 & \cdots & 0 & a_{11} & \cdots & a_{1N} \\ \vdots & \ddots & \vdots & \vdots & & \vdots \\ 0 & \cdots & -1 & a_{N1} & \cdots & a_{NN} \\ 1 & \cdots & 0 & -\gamma_1 & \cdots & 0 \\ \vdots & \ddots & \vdots & \vdots & \ddots & \vdots \\ 0 & \cdots & 1 & 0 & \cdots & -\gamma_N \end{pmatrix}$$

Therefore, for a system of N interacting species, the Jacobian is a $2N \times 2N$ matrix whereby the top right quadrant, which corresponds to the derivative of the unspliced force field with respect to spliced species (highlighted in red box), assumes the interpretation of a gene regulatory network.

2. Generation of in silico data

<u>2.1 Toggle switch</u>

We generalize a minimal toggle switch design with two species (x, y) that mutually inhibit each other to include splicing dynamics while conserving the model's bistability. The temporal dynamics of unspliced and spliced versions of x and y (U_x, U_y, S_x, S_y) is modeled via stochastic ordinary differential equations:

$$\frac{dU_x}{dt} = \frac{\alpha}{1 + {S_y}^{\beta_1}} - \gamma U_x + \sigma\, dW(t) \quad (1a)$$
$$\frac{dU_y}{dt} = \frac{\alpha}{1 + {S_x}^{\beta_2}} - \gamma U_y \ + \sigma\, dW(t) \quad (1b)$$
$$\frac{dS_x}{dt} = \gamma U_x - S_x + \sigma\, dW(t) \quad (1c)$$
$$\frac{dS_y}{dt} = \gamma U_y - S_y + \sigma\, dW(t) \quad (1d)$$

Where α is a basal production rate, γ is a splicing rate coefficient, and β_1 , β_2 are Hill coefficients that determine the strength of feedback regulation. To simplify the model, α and γ are assumed to be equal for the two species. Furthermore, the degradation/dilution coefficient for spliced species S_x, S_y is assumed to be 1, which can be achieved by rescaling time. Finally, the noise $\sigma\, dW(t)$ is the product of an amplitude term σ and a random variable sampled from a normal distribution with standard deviation $\sqrt{\Delta t}$, where Δt is the timestep used for numerical solution. To induce a saddle node bifurcation, the parameter β_1 is gradually increased with time starting from $\beta_1 = 2$ to $\beta_1 = 15$. To generate the *in silico* dataset, we simulated $n = 1000$ trajectories of eqs. (1) using Euler-Maruyama starting from $t = 0$ (i.e., $\beta_1 = 2$) until $t = 200$ (i.e., $\beta_1 = 15$). From each independent simulation, a "cell" is sampled by taking the values of (U_x, U_y, S_x, S_y) at a randomly selected point along the trajectory. All other parameter values are fixed to $\alpha = 1$, $\beta_2 = 6$, $\gamma = 1$, $\sigma = 1$. All code was generated by the authors.

2.2 EMT circuit

We simulate epithelial-mesenchymal transition (EMT) with the EMT tristable circuit proposed by Tian and collaborators, which we previously generalized to include RNA splicing dynamics. This model uses ODEs to describe the dynamics of unspliced and spliced levels for: miR-34, miR-200, ZEB, SNAIL, external TGF-beta signal, cellular TGF-beta, E-cadherin, and N-cadherin. For each species, the unspliced equation includes basal transcription rate, feedback regulation from other species, and loss due to splicing; the spliced equation includes production due to splicing and degradation/dilution:

Intracellular TGF-beta (U_T, S_T):

$$\frac{dU_T}{dt} = k0_T + \frac{k_T}{1 + \left(\frac{S_{R_2}}{J_T}\right)^{n_{r2}}} - \beta U_T \quad (4a)$$
$$\frac{dS_T}{dt} = \beta U_T - kd_T S_T \quad (4b)$$

Snail mRNA (U_s, S_s) and protein (U_S, S_S):

$$\frac{dU_s}{dt} = k0_s + k_s \frac{\left(\frac{S_T + TGF0}{J_T}\right)^{n_t}}{1 + \left(\frac{S_T + TGF0}{J_T}\right)^{n_t}} - \beta U_s \quad (5a)$$
$$\frac{dS_s}{dt} = \beta U_s - kd_s S_s \quad (5b)$$

$$\frac{dU_S}{dt} = k0_s + k_S S_s \frac{1}{1 + \left(\frac{S_{R_3}}{J_S}\right)^{n_{r3}}} - \beta U_S \quad (6a)$$

$$\frac{dS_S}{dt} = \beta U_S - kd_S S_S \quad (6b)$$

miR-34 (U_{R3}, S_{R3}):

$$\frac{dU_{R3}}{dt} = k0_3 + \frac{k_3}{1 + \left(\frac{S_S}{J1_3}\right)^{n_S} + \left(\frac{S_Z}{J2_3}\right)^{n_Z}} - \beta U_{R3} \quad (7a)$$

$$\frac{dS_{R3}}{dt} = \beta U_{R3} - kd_3 S_{R3} \quad (7b)$$

Zeb mRNA (U_z, S_z) and protein (U_Z, S_Z):

$$\frac{dU_z}{dt} = k0_z + k_z \frac{\left(\frac{S_S}{J_Z}\right)^{n_s}}{1 + \left(\frac{S_S}{J_Z}\right)^{n_s}} - \beta U_z \quad (8a)$$

$$\frac{dS_z}{dt} = \beta U_z - kd_z S_z \quad (8b)$$

$$\frac{dU_Z}{dt} = k0_Z + k_Z S_z \frac{1}{1 + \left(\frac{S_{R_2}}{J_Z}\right)^{n_{r2}}} - \beta U_Z \quad (9a)$$

$$\frac{dS_Z}{dt} = \beta U_Z - kd_Z S_Z \quad (9b)$$

miR-200 (U_{R2}, S_{R2}):

$$\frac{dU_{R2}}{dt} = k0_2 + \frac{k_2}{1 + \left(\frac{S_S}{J1_2}\right)^{n_S} + \left(\frac{S_Z}{J2_2}\right)^{n_Z}} - \beta U_{R2} \quad (10a)$$

$$\frac{dS_{R2}}{dt} = \beta U_{R2} - kd_2 S_{R2} \quad (10b)$$

E-cadherin (U_E, S_E):

$$\frac{dU_E}{dt} = k0_E + \frac{k_{E1}}{1 + \left(\frac{S_S}{J1_E}\right)^{n_S}} + \frac{k_{E2}}{1 + \left(\frac{S_Z}{J2_E}\right)^{n_Z}} - \beta U_E \quad (11a)$$

$$\frac{dS_E}{dt} = \beta U_E - kd_E S_E \quad (11b)$$

N-cadherin (U_N, S_N):

$$\frac{dU_N}{dt} = k0_N + k_{N1} \frac{\left(\frac{S_S}{J1_N}\right)^{n_S}}{1 + \left(\frac{S_S}{J1_N}\right)^{n_S}} + k_{N2} \frac{\left(\frac{S_Z}{J2_N}\right)^{n_Z}}{1 + \left(\frac{S_Z}{J2_N}\right)^{n_Z}} - \beta U_N \quad (12a)$$

$$\frac{dS_N}{dt} = \beta U_N - kd_N S_N \quad (12b)$$

Parameter values were taken from the original model. The splicing rate coefficient (β) is assumed to be rescaled to unit value. The system of ODEs was simulated numerically using Euler-Maruyama algorithm. All code was generated by the authors.

2.3 Trifurcating circuit

We simulate $n = 2000$ cells in the trifurcating circuit consisting of 8 mutually regulating genes using the BoolODE package. All circuit and simulation parameter were fixed to their original value. The output of BoolODE consists of two gene expression matrices of size 8x2000 corresponding to two layers of expression, interpreted here as the unspliced and spliced RNA counts, and the simulation time associated to each cell.

3. Calculation and visualization of instability score

The instability score of a single cell along a lineage is defined as the average number of positive eigenvalues associated with the cell. Since a cell can appear in multiple bins along pseudotime axis (see previous section), the score averages the number of positive eigenvalues in each pseudotime bin where the cell is found. When multiple lineages are present in the dataset (see Fig. 3), the score is first computed within each individual lineage and then averaged if the cell belongs to multiple lineages. Finally, the instability score is plotted and overlayed on RNA velocity using scVelo's velocity_embedding_stream() function.

4. Data preparation and clustering

The unspliced and spliced scRNA-seq RNA counts for both OVCA420 and A549 datasets were taken from Cook and collaborators, and later filtered and normalized following standard procedures using scanpy. The n=50 top genes were used for Jacobian inference and downstream GRN analysis. Diffusion pseudotime for the OVCA420 dataset was computed using scanpy's built-in diffusion pseudotime function with default parameters, whereby the root cell for pseudotime calculation (i.e., the cell at pseudotime t=0) was set as a randomly chosen cell from the cell group sampled at t=0 days. Velocity pseudotime for the A549 dataset was computed using scVelo's built in function with default parameters.

5. Gene selection criteria

Scanpy's highly variable feature function was used to select the top 50 differentially expressed genes for both the OVCA420 and A549 cell line datasets. The set of top 50 genes used in Figure 4 are presented in supplementary table 2. Furthermore, the number of differentially expressed genes selected via scanpy's highly variable feature function was varied (N=25, 50, 75, 100) to test the method robustness (supplementary figure S5). Finally, we test the method using a user-curated list of epithelial and mesenchymal genes recovered from literature. First, we used scanpy's highly variable feature function to select the top 2000 genes in the dataset to ensure that low-count genes were filtered out. Then, we subset the dataset and kept only the epithelial and mesenchymal genes, resulting in 24 epithelial genes and 60 mesenchymal genes (supplementary table 3).

6. Inference of Jacobian matrix and gene regulatory network

The input for Jacobian inference includes the unspliced and spliced RNA counts as well as the pseudotime value for each cell defined in the previous section. First, cells were binned within intervals along the pseudotime coordinate. A width parameter (w) sets how wide the pseudotime

bins are, whereas an increment parameter (inc) sets the forward step taken along the pseudotime axis after each inference iteration. Considering that diffusion pseudotime is typically bound in [0,1], we selected w=0.1, inc=0.05, leading to 20 equally spaced points along the lineage. We stress that a "correct" value for these parameters cannot be established a priori, and it is the result of trial and error. Second, all cells within each bin are used to infer the Jacobian matrix and gene regulatory network following the strategy highlighted in the main method section "*Identification of tipping points along lineage*".

7. Comparison with Dynamo

While our method computes an average Jacobian matrix within a specified reaction coordinate window (diffusion pseudotime in the case of Fig. 4), Dynamo reconstructs a Jacobian matrix associated to individual cells. To set up a fair comparison, we average the Jacobian matrix calculated by Dynamo over the same pseudotime inference window. Initially, we run Dynamo's Vectorfield function to compute a cell-specific Jacobian matrix. Then, we group cells based on their position along the diffusion pseudotime axis, and average Dynamo's cell-specific Jacobian matrices. We compare the methods based on different criteria for gene set selection. First, we let Dynamo's preprocessing pipeline select the genes for Jacobian inference; second, we filter the dataset object with the genes used in our calculation before running Dynamo's Vectorfield function. Third, we provide the gene set used in our calculation as input for Dynamo's Vectorfield function. In all three cases, the stability along the EMT lineage is evaluated based on both (1) The number of positive eigenvalues and (2) the value of the largest eigenvalue.

8. Community detection in gene regulatory network

GRN graph objects were generated using the NetworkX package. GRN communities were calculated using NetworkX's built-in functions girvan_newman() and greedy_modularity_communities() using default parameters.

9. Code availability

All scripts were generated in by the authors and are available at https://github.com/barcenasmanuel/Detecting_bifurcations_in_scRNA_seq_data.

OVCA420	TSPAN1, ATP1B1, LAMC2, CENPF, GATA3, PLAU, FGF19, LIMA1, KRT81, KRT6A, HSP90B1, NFKBIA, SNAPC1, C15orf48, NQO1, MMP2, CCL5, TOP2A, BCAS3, KPNA2, SIPA1L3, CCDC88A, SERPINE2, ID2, CENPA, ANXA4, DHRS9, TGM2, AURKA, MMP9, CBR1, CBR3, LGALS1, MYLK, SPTSSB, TPRG1, C4orf26, ANXA3, ANXA10, FGF1, CDH6, FST, TFPI2, PODXL, HSPB1, SLC7A2, LPAR1, AMBP, TNC, SAT1

Supplementary table 1. List of top 50 genes used for stability inference in in OVCA420 cell line (Figure 4).

Epithelial geneset	ALDH1A3, AREG, DST, CD9, CDH1, EMP1, EPAS1, EREG, KRT19, ABLIM1, MITF, ATP8B1, PKP2, PPL, NR2F2, TPD52L1, MAP7, ARHGAP29, TJP2, HS3ST1, TSPAN1, SYNE2, EHF, GULP1
Mesenchymal geneset	ACTN1, BMPR2, BPGM, CALD1, CD59, CDH2, COL4A1, COL4A2, ELK3, EPHB2, ETS2, FN1, HMOX1, JARID2, LAMC2, MMP2, MMP9, MYO10, NEDD9, NT5E, SERPINE1, SERPINE2, PODXL, PTHLH, PTPRK, RALA, SKIL, TGFB1, TGFBI, TGM2, THBS1, TPM1, TPM4, TUFT1, TFPI2, HMGA2, INPP4B, PDLIM7, TP53I3, MICAL2, DLC1, SEMA3C, DAAM1, PALLD, TMCC1, PLEK2, DSE, LMCD1, CHST11, ANGPTL4, GALNT10, PDGFC, PMEPA1, SMURF2, FHOD3, LBH, MAP1LC3B, MBOAT2, AMIGO2, VGLL3

Supplementary Table 2. List of epithelial and mesenchymal genes used to compute mesenchymal scores (Fig. 4B-C and 5B-C) and the Jacobian inference based on the EMT gene signature.

Supplementary Figures

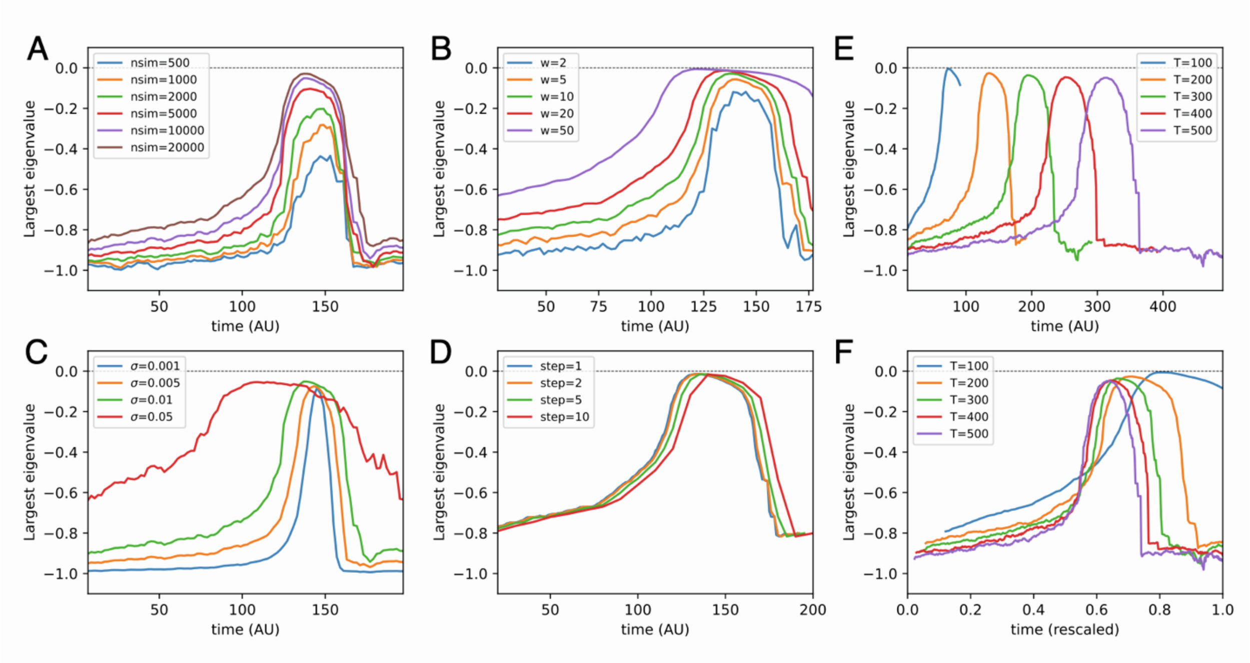

Supplementary figure S1. Supplementary analysis of the toggle switch circuit. (A) *Largest eigenvalue inferred in the toggle switch Jacobian matrix as a function of simulation time for increasing number of sampled cells.* ***(B)*** *Same as (A) for varying width of the inference window parameter.* ***(C)*** *Same as (A) for varying levels of noise amplitude.* ***(D)*** *Same as (A) for varying increment step of the inference window parameter.* ***(E)*** *Largest eigenvalue of the toggle switch Jacobian matrix as a function of time for increasing simulation length. The shortest simulation length (T=100 AU, blue curve) is not sufficient for cells to complete the saddle-node transition.* ***(F)*** *Same as (E) when renormalizing time (x-axis). The toggle switch undergoes the saddle-node transition at similar rescaled time for all simulation length except T=100 AU. Foe each panel, default parameters are same as in main Figure 2E-F (nsim=2000, σ=0.005, w=10, step=2, T=200 AU) except for the parameter that is varied in the specific panel.*

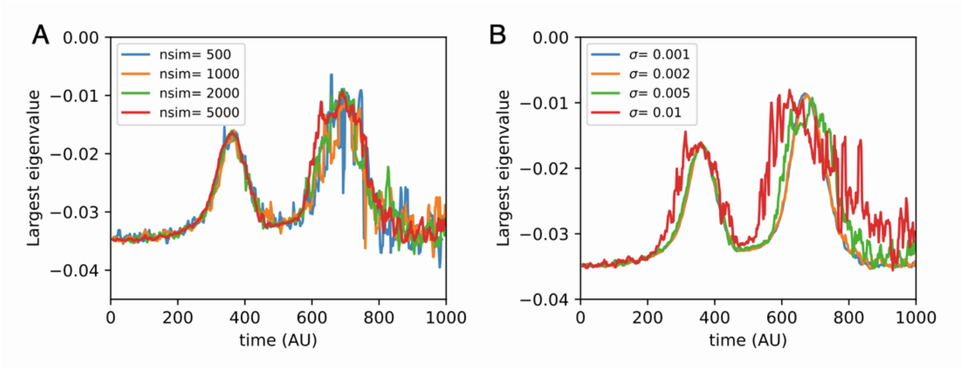

Supplementary figure S2. Supplementary analysis of the EMT circuit. (A) *Largest eigenvalue inferred in the EMT Jacobian matrix as a function of simulation time for increasing number of sampled cells.* ***(B)*** *Same as (A) for varying levels of noise amplitude. Foe each panel, default parameters are same as in main Figure 2G-H (nsim=2000, σ=0.005, w=10, step=2, T=1000 AU) except for the parameter that is varied in the specific panel.*

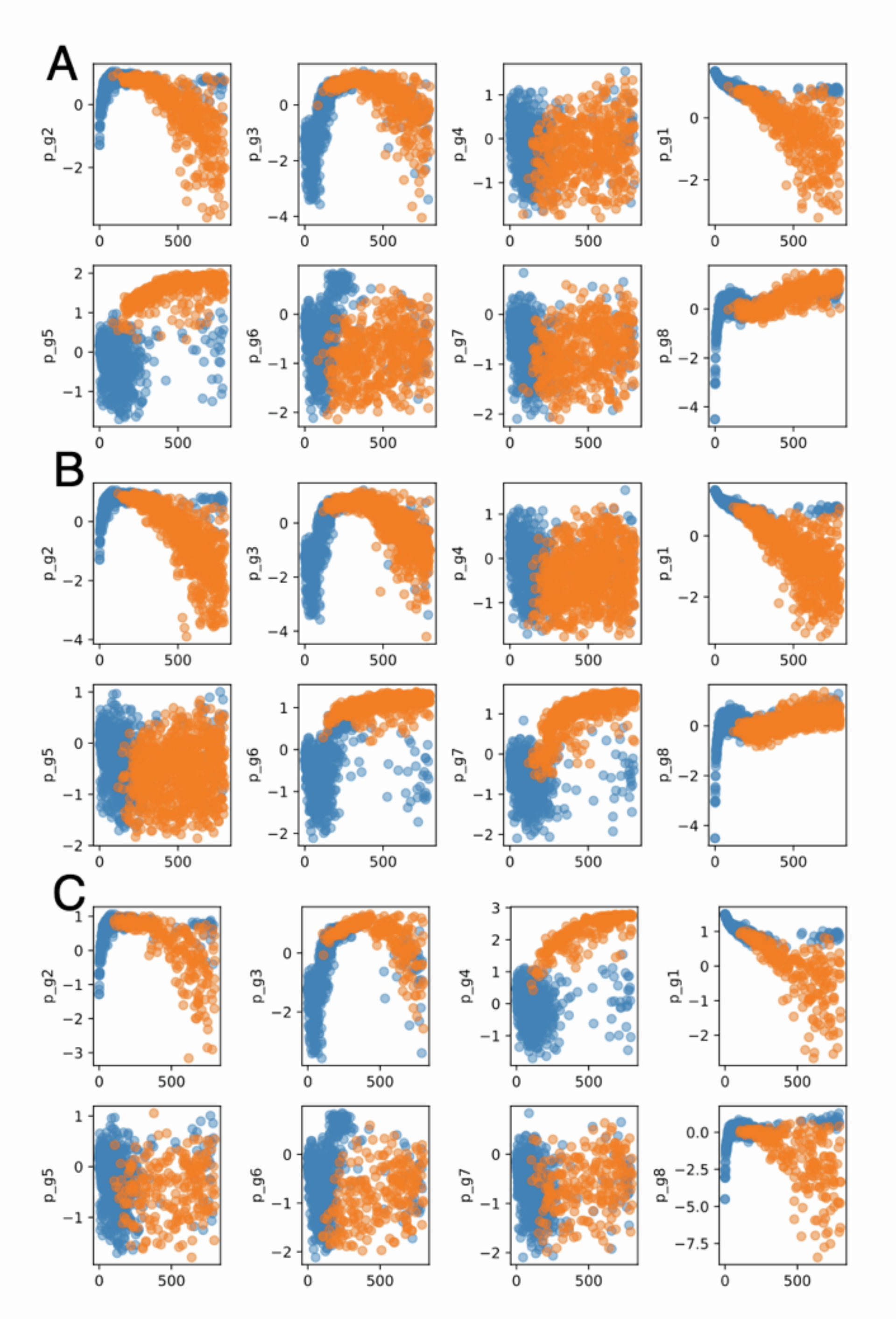

Supplementary figure S3. Supplementary analysis of the trifurcating circuit. (A) *Dynamics of the 8 spliced variable in the S-T1 transition in the trifurcating circuit. Each dot represents a simulated cell plotted against BoolODE simulation time (x-axis). Orange and blue coloring indicates cells belonging to the S and T1 clusters, respectively.* ***(B-C)*** *Same as (A) for the S-T2 and S-T3 transitions, respectively.*

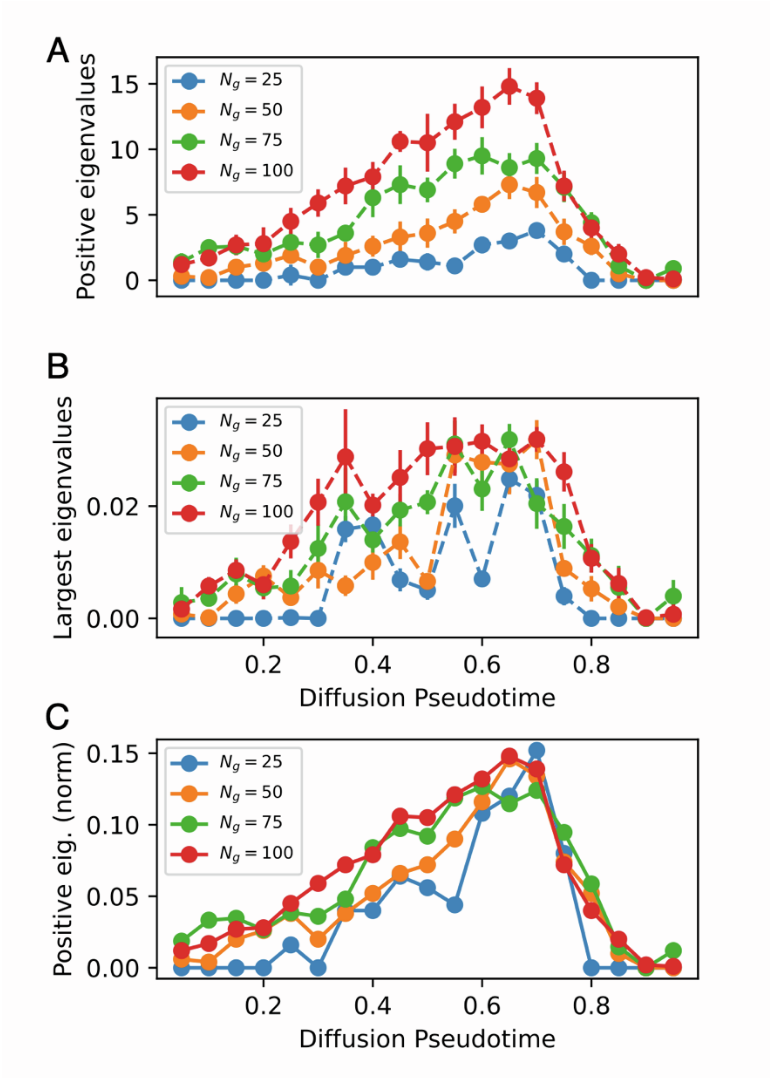

Supplementary figure S4. Method robustness to variation of the number of genes. (A) *Number of positive Jacobian eigenvalues as a function of diffusion pseudotime in the OVCA420 cell line dataset for increasing number of top genes selected for Jacobian inference.* ***(B)*** *Largest Jacobian eigenvalue as a function of pseudotime for increasing number of top genes.* ***(C)*** *Number of positive eigenvalues normalized by the total number of selected genes.*

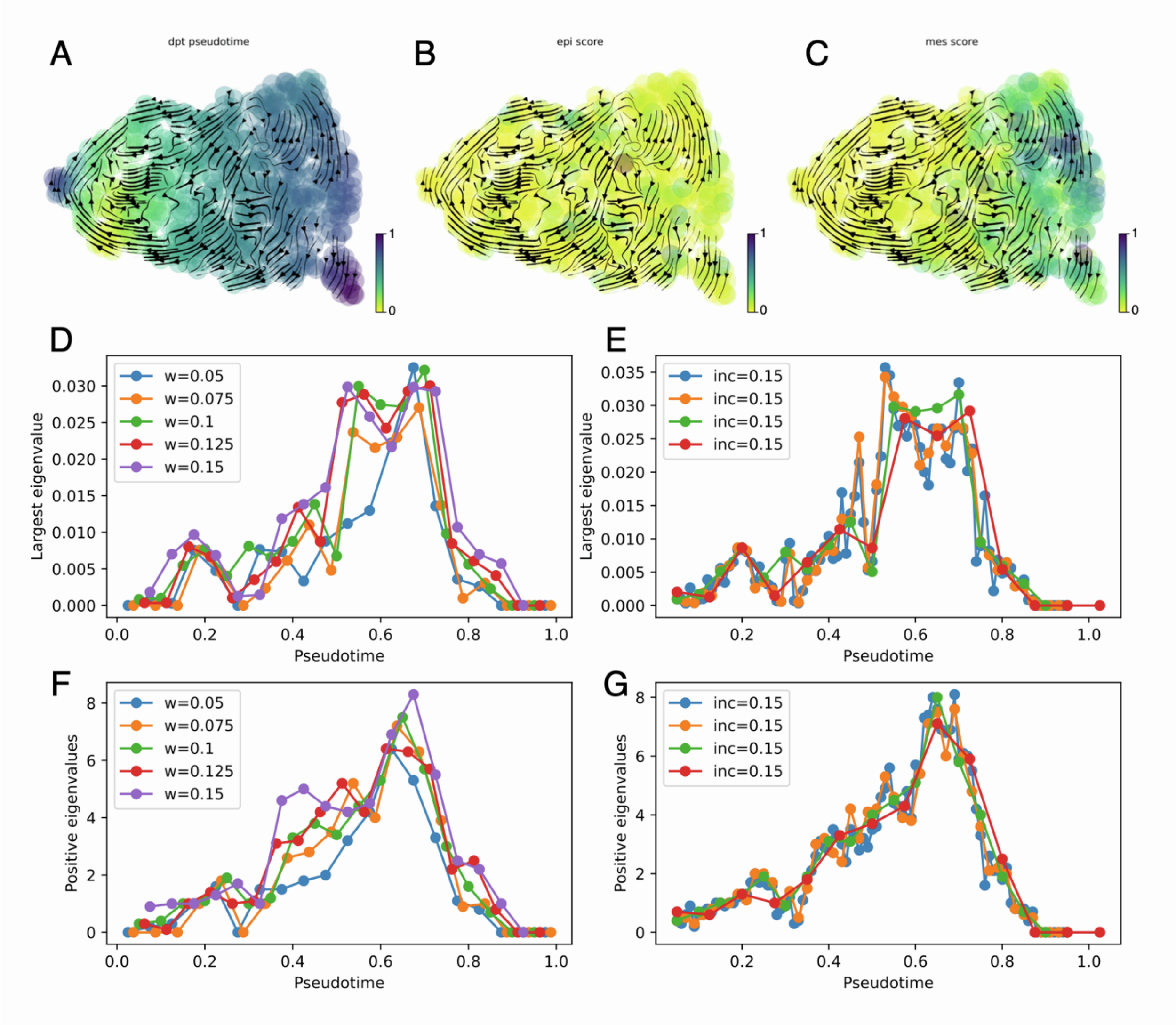

Supplementary figure S5. Supplementary analysis of the OVCA420 dataset. (A-B-C) *Diffusion pseudotime (A), epithelia score (B), and mesenchymal score (C) of OVCA420 cells overlayed on UMAP and RNA velocity.* ***(D)*** *Largest eigenvalue as a function of diffusion pseudotime. Different curves highlight different values of inference window width expressed in pseudotime units.* ***(E)*** *Largest eigenvalue as a function of diffusion pseudotime upon variation of inference window increment, expressed in pseudotime units.* ***(F-G)*** *Same as (D-E) for number of positive eigenvalues as a function of pseudotime.*

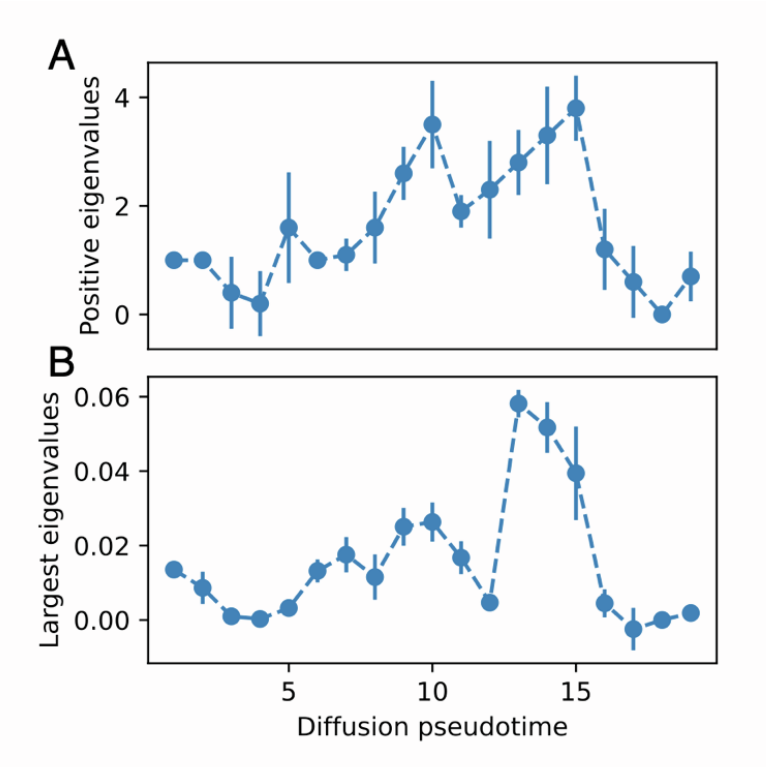

Supplementary figure S6. Jacobian inference based on EMT gene set. (A-B) *Number of positive eigenvalues (A) and largest Jacobian eigenvalue (B) in the OVCA420 cell line dataset as a function of diffusion pseudotime when a set of epithelial and mesenchymal genes is used for Jacobian inference.*

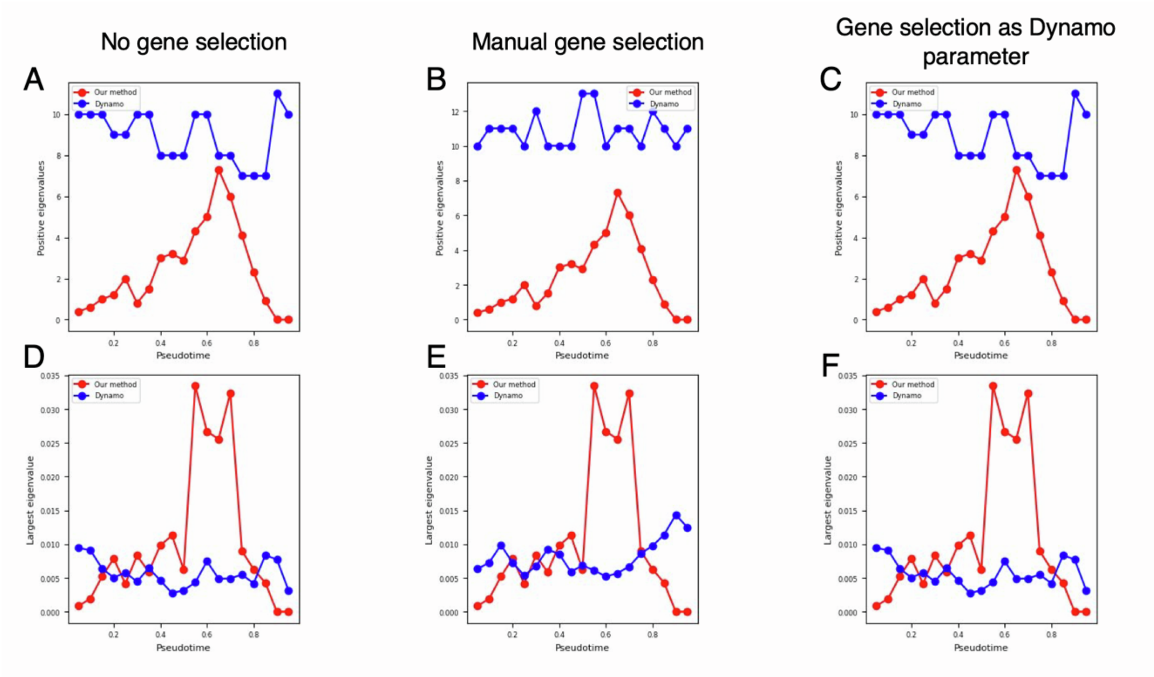

Supplementary figure S7. Comparison with Dynamo. (A-B) *Comparison for number of positive eigenvalues (A) and largest Jacobian eigenvalue (B) in the OVCA420 cell line dataset as a function of diffusion pseudotime between our method (red) and Dynamo (blue) when default criteria for Dynamo's gene selection. (C-D) Same as (A-B) when the dataset is manually filtered to include the top 50 genes used in our analysis before Dynamo is run. (E-F) Same as (A-B) when the list of top 50 genes used in our calculation is passed to Dynamo's Vectorfield function.*

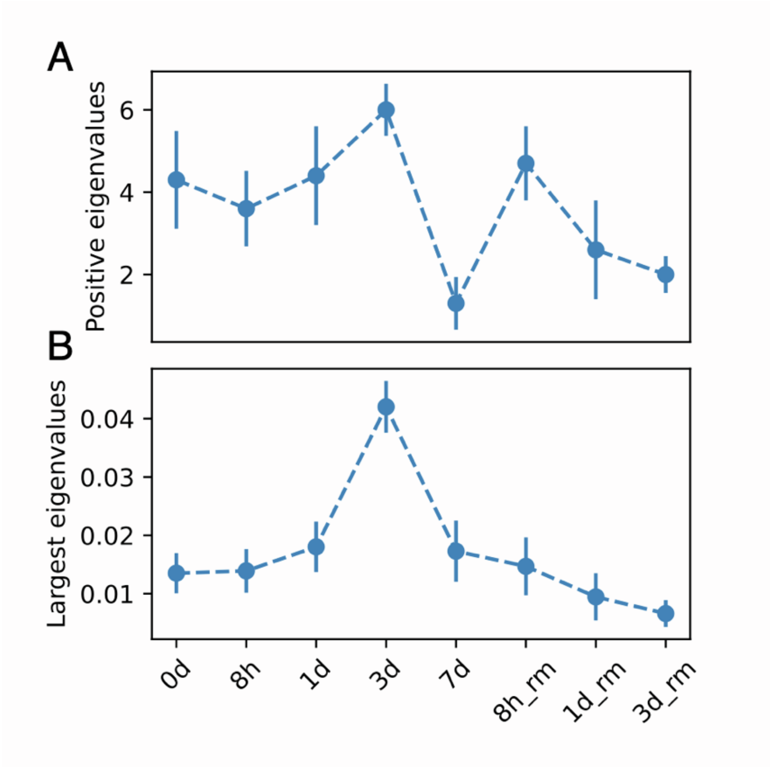

Supplementary figure S8. Jacobian inference based on time course points. (A-B) *Number of positive eigenvalues (A) and largest Jacobian eigenvalue (B) in the OVCA420 cell line dataset when using the time points if the time course for Jacobian inference.*

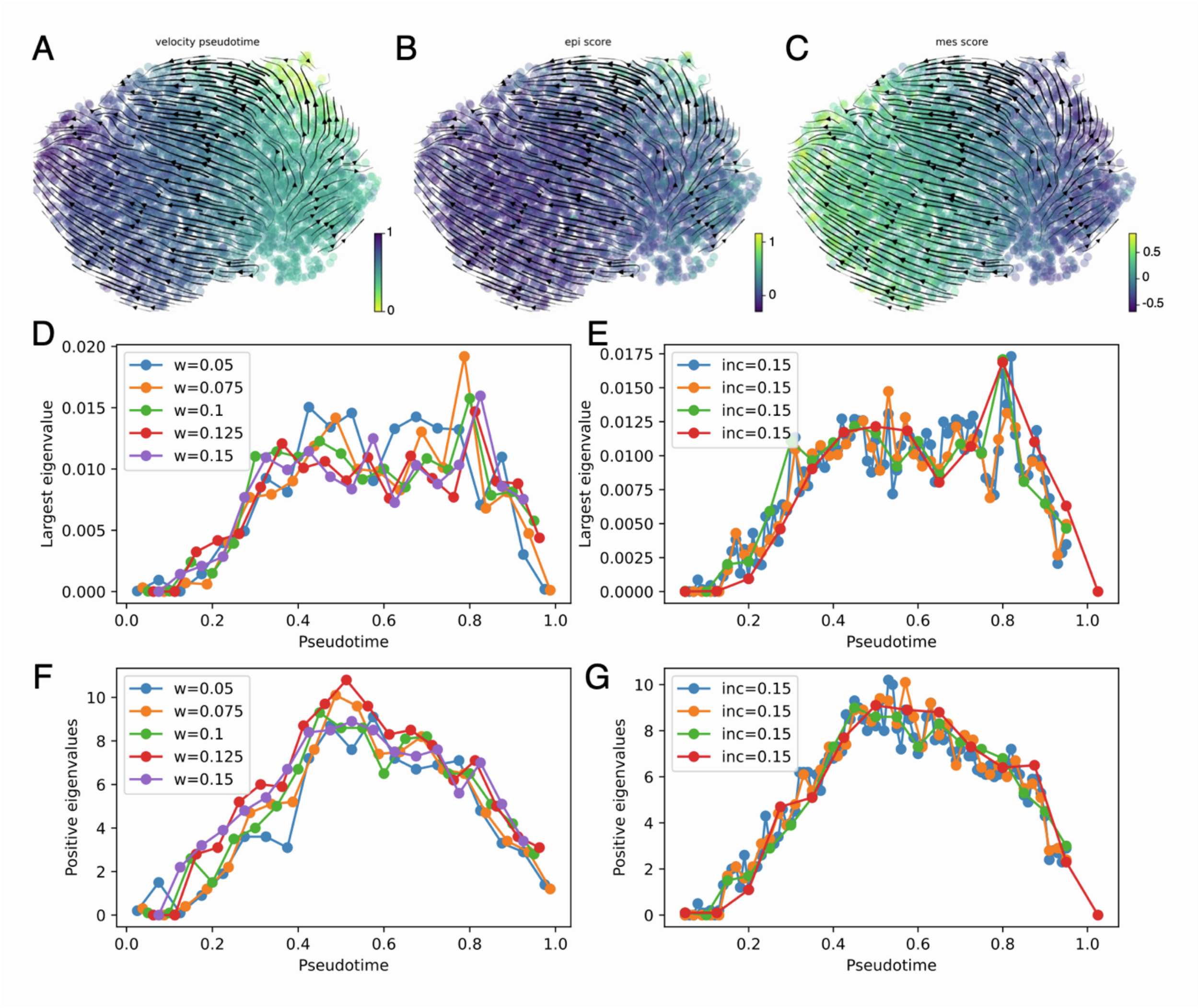

Supplementary figure S9. Supplementary analysis of the A549 dataset. (A-B-C) *Diffusion pseudotime (A), epithelia score (B), and mesenchymal score (C) of A549 cells overlayed on UMAP and RNA velocity.* ***(D)*** *Largest eigenvalue as a function of diffusion pseudotime. Different curves highlight different values of inference window width expressed in pseudotime units.* ***(E)*** *Largest eigenvalue as a function of diffusion pseudotime upon variation of inference window increment, expressed in pseudotime units.* ***(F-G)*** *Same as (D-E) for number of positive eigenvalues as a function of pseudotime.*

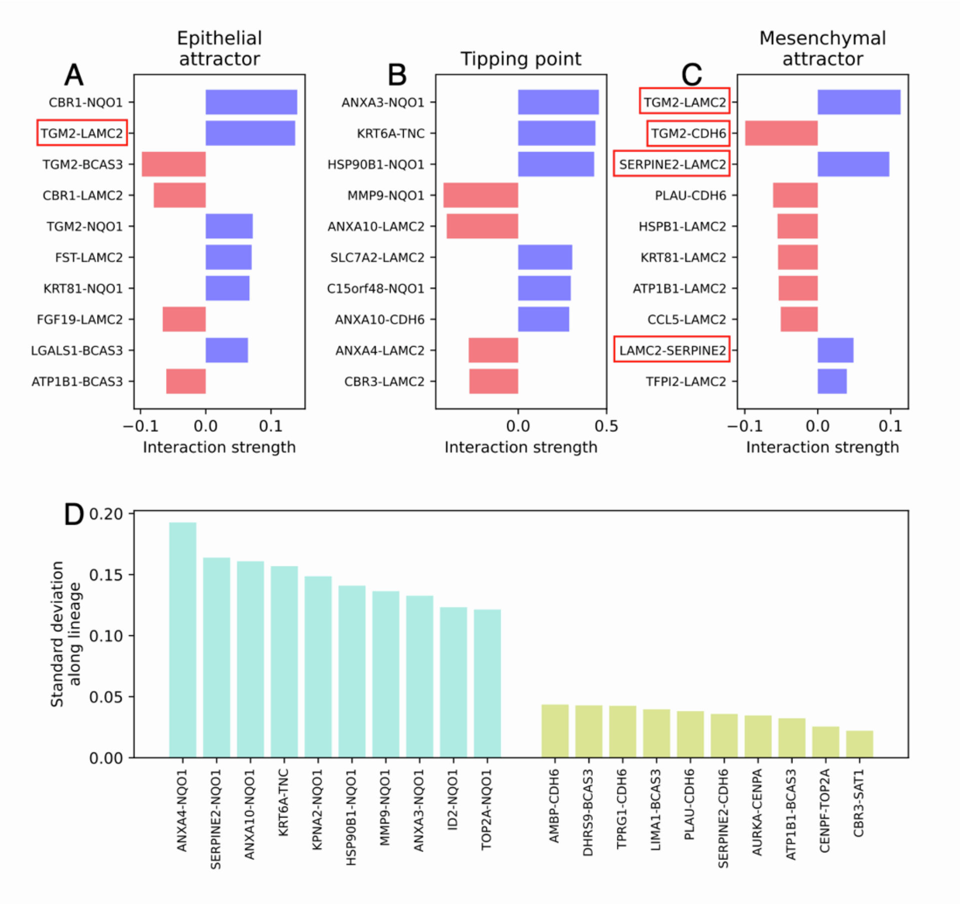

Supplementary figure S10. Statistics of gene-gene interactions in OVCA420 cell line. *(A-B-C) The top 10 strongest gene-gene interactions inferred in the pseudotime points corresponding to the epithelial attractor (as in Fig. 5B), tipping point (Fig. 5D), and mesenchymal attractor (Fig. 5F). Red boxes highlight connections between known epithelial (CHD6) and mesenchymal (TGM2, LAMC2, SERPINE2) genes showing that mesenchymal genes tend to positively regulate each other while inhibiting CDH6. (D) Statistics of the gene-gene interactions with larger (left, cyan) and lowest (right, yellow) standard deviation along the lineage.*
